# Adaptive formation learning control for cooperative AUVs under complete uncertainty

**DOI:** 10.3389/frobt.2024.1491907

**Published:** 2025-02-14

**Authors:** Emadodin Jandaghi, Mingxi Zhou, Paolo Stegagno, Chengzhi Yuan

**Affiliations:** ^1^ Department of Mechanical, Industrial and Systems Engineering, University of Rhode Island, Kingston, RI, United States; ^2^ Graduate School of Oceanography, University of Rhode Island, Kingston, RI, United States; ^3^ Department of Electrical, Computer, and Biomedical Engineering, University of Rhode Island, Kingston, RI, United States

**Keywords:** environment-independent controller, autonomous underwater vehicles (AUV), dynamic learning, formation learning control, multi-agent systems, neural network control, adaptive control, robotics

## Abstract

**Introduction:**

This paper addresses the critical need for adaptive formation control in Autonomous Underwater Vehicles (AUVs) without requiring knowledge of system dynamics or environmental data. Current methods, often assuming partial knowledge like known mass matrices, limit adaptability in varied settings.

**Methods:**

We proposed two-layer framework treats all system dynamics, including the mass matrix, as entirely unknown, achieving configuration-agnostic control applicable to multiple underwater scenarios. The first layer features a cooperative estimator for inter-agent communication independent of global data, while the second employs a decentralized deterministic learning (DDL) controller using local feedback for precise trajectory control. The framework's radial basis function neural networks (RBFNN) store dynamic information, eliminating the need for relearning after system restarts.

**Results:**

This robust approach addresses uncertainties from unknown parametric values and unmodeled interactions internally, as well as external disturbances such as varying water currents and pressures, enhancing adaptability across diverse environments.

**Discussion:**

Comprehensive and rigorous mathematical proofs are provided to confirm the stability of the proposed controller, while simulation results validate each agent’s control accuracy and signal boundedness, confirming the framework’s stability and resilience in complex scenarios.

## 1 Introduction

Robotics and autonomous systems have a wide range of applications, spanning from manufacturing and surgical procedures to exploration in challenging environments (Ghafoori et al., 2024; Jandaghi et al., 2023). However, controlling robots in such settings, especially in space and underwater, presents significant difficulties due to unpredictable dynamics. In the context of underwater exploration, AUVs have become essential tools, offering cost-effective, reliable, and versatile solutions for adapting to dynamic conditions. Effective use of AUVs is critical for unlocking the mysteries of marine environments, making advancements in their control and operation essential. As the demand for efficient underwater exploration increases and the complexity of tasks assigned to AUVs grows, there is a pressing need to enhance their operational capabilities. This includes developing sophisticated formation control strategies that allow multiple AUVs to operate in coordination, drawing inspiration from natural behaviors observed in fish schools and bird flocks (Zhou et al., 2023; Yang et al., 2021). By leveraging multi-agent systems, AUVs can work in coordinated groups, enhancing efficiency, stability, and coverage while navigating dynamic and complex underwater environments. These strategies are essential for ensuring precise operations in varied underwater tasks, ranging from pipeline inspections and seafloor mapping to environmental monitoring (Yan et al., 2023).

Despite challenges from intricate nonlinear dynamics, complex interactions among AUVs, and the uncertain dynamic nature of underwater environments, effective multi-AUV formation control is increasingly critical in modern ocean industries (Yan et al., 2018; Hou and Cheah, 2009). Historically, formation control research has predominantly utilized the behavioral approach (Balch and Arkin, 1998; Lawton, 2000), which divides the overall control design into subproblems, with each vehicle’s action determined by a weighted average of solutions, though selecting appropriate weighting parameters can be challenging. The leader-following approach (Cui et al., 2010; Rout and Subudhi, 2016) designates one vehicle as the leader while others follow, maintaining predefined geometric relationships, and controlling formation behavior by designing specific motions for the leader. Alternatively, the virtual structure approach (Millán et al., 2013).

Despite advancements in formation control and path planning for multi-AUV systems, challenges such as environmental disturbances, complex underwater dynamics, and communication limitations continue to pose difficulties (Hadi et al., 2021). To address these challenges, there is a critical need for controllers that are independent of both robot dynamics and environmental disturbances. Developing such controllers would enhance formation control by allowing for decentralized application, which increases flexibility in formation structures and improves robustness against communication constraints. Addressing these gaps is essential for advancing the capabilities and reliability of multi-AUV systems. On the other hand, communication constraints in underwater environments make decentralized control with a virtual leader-following topology ideal for AUVs, enabling coordination using local information despite communication delays or interruptions (Yan et al., 2023).

Reinforcement learning (RL) has also been extensively applied in robotic control (Christen et al., 2021; Cao et al., 2022). RL approaches, such as deep reinforcement learning (DRL), offer advantages in learning complex, non-linear control policies directly from data. However, RL methods generally lack the ability to provide mathematical stability proofs and guarantees for the controller’s behavior, making it challenging to ensure safety and reliability, especially in critical applications. Besides, while Zhang et al. (2018) developed various direct neural adaptive laws that lead to increased oscillations with higher adaptation gains, indirect neural adaptive laws using prediction error methods were proposed to mitigate this issue, though they could not guarantee parameter convergence. However, NN-based learning control methods, such as those utilizing adaptive neural networks or deterministic learning frameworks Jandaghi et al. (2024), can incorporate stability analysis and provide rigorous mathematical proofs for parameter convergence. These methods enable researchers to establish theoretical guarantees for the stability and robustness of the controller, which is essential for deploying controllers in real-world applications where safety and reliability are critical. Most recently, Tutsoy et al. (2024) proposed an optimization-based approach for path planning in Unmanned Air Vehicles (UAVs) with actuator failures using particle swarm optimization and genetic algorithms. Their method focuses on minimizing both time and distance by optimizing predefined cost functions through heuristic methods, while incorporating system constraints such as actuator limits, kinematic, and dynamic constraints, as well as parametric uncertainties.

Despite extensive literature in the field, to the best of our knowledge, existing researches assume homogeneous dynamics and certain system parameters for all AUV agents, which is unrealistic in unpredictable underwater environments. Factors such as buoyancy, drag, and varying water viscosity significantly alter system dynamics and behavior. Additionally, AUVs may change shape during tasks like underwater sampling or when equipped with robotic arms, further complicating control. Typically, designing multi-AUV formation control involves planning desired formation paths and developing tracking controllers for each AUV. However, accurately tracking these paths is challenging due to the complex nonlinear dynamics of AUVs, especially when precise models are unavailable. Implementing a fully distributed and decentralized formation control system is also difficult, as centralized control designs become exceedingly complex with larger AUV groups. To address these challenges previous work, such as Yuan et al. (2017) and Dong et al. (2019), developed adaptive learning controllers that relied on the assumption of a known mass matrix, which is not practical in real-world applications. These controllers relied on known system parameters that can fail due to varying internal forces caused by varying external environmental conditions. The solution is to develop environment-independent controllers that do not rely on any specific system dynamical parameters.

The framework’s control architecture is ingeniously divided into a first-layer Cooperative Estimator Observer and a lower-layer Decentralized Deterministic Learning (DDL) Controller. The first-layer observer is pivotal in enhancing inter-agent communication by sharing crucial system estimates, operating independently of any global information. Concurrently, the second-layer DDL controller utilizes local feedback to finely adjust each AUV’s trajectory, ensuring resilient operation under dynamic conditions heavily influenced by hydrodynamic forces and torques by considering system uncertainty completely unknown. This dual-layer setup not only facilitates acute adaptation to uncertain AUV dynamics but also leverages RBFNN for precise local learning and effective knowledge storage. Such capabilities enable AUVs to efficiently reapply previously learned dynamics after the system restarts. This tow-layer framework achieves a significant advancement by considering all system dynamics parameters as unknown, enabling a universal application across all AUVs, regardless of their operating environments. This universality is crucial for adapting to environmental variations such as water flow, which increases the AUV’s effective mass via the added mass phenomenon and affects the vehicle’s inertia. Additionally, buoyancy forces that vary with depth, along with hydrodynamic forces and torques, stemming from water flow variations, the AUV’s unique shape, its appendages, and drag forces due to water viscosity, significantly impact the damping matrix in the AUV’s dynamics. This framework not only improves operational efficiency but also significantly advances the field of autonomous underwater vehicle control by laying a robust foundation for future enhancements in distributed adaptive control systems and fostering enhanced collaborative intelligence among multi-agent networks in marine environments. Extensive simulations have underscored the effectiveness of the framework, demonstrating its potential to elevate the adaptability and resilience of AUV systems under the most demanding conditions. In summary, the contribution of this paper is as follows:

• The universal controller works in any environment and condition, such as currents or depth.
• Each AUV controller operates independently.
• The controller functions without needing information about the robot’s dynamic parameters, like mass, damping, or inertia. Each AUV can also have different dynamic parameters.
• The system learns the dynamics once and reuses the pre-trained weights, avoiding the need for retraining.
• The use of localized RBFNN reduces real-time computational demands.
• Providing rigorous stability analysis of the controller while providing mathematical proofs to ensure and guarantee the reliability of the controller.

The rest of the paper is organized as follows: Section 2 provides an initial overview of graph theory, RBFNN, and the problem statement. The design of the distributed cooperative estimator and the decentralized deterministic learning controller are discussed in Section 3. The formation adaptive control and formation control using pre-learned dynamics are explored in Section 4 and Section 5, respectively. Simulation studies are presented in Section 6, and Section 7 concludes the paper.

## 2 Preliminaries and problem statement

### 2.1 Notation and graph theory

Denoting the set of real numbers as R , we define $R^{m\times n}$ as the set of m×n real matrices, and $R^{n}$ as the set of n×1 real vectors. The identity matrix is symbolized as I . The vector with all elements being 1 in an n -dimensional space is represented as $1_{n}$ . The sets $S_{+}^{n}$ and $S_{-}^{n+}$ stand for real symmetric n×n and positive definite matrices, respectively. A block diagonal matrix with matrices $X_{1},X_{2},\ldots ,X_{p}$ on its main diagonal is denoted by $\text{diag}\left\{X_{1},X_{2},\ldots ,X_{p}\right\}$ . A⊗B signifies the Kronecker product of matrices A and B . For a matrix A , $\vec{A}$ is the vectorization of A by stacking its columns on top of each other. For a series of column vectors $x_{1},\ldots ,x_{n}$ , $\text{col}\left\{x_{1},\ldots ,x_{n}\right\}$ represents a column vector formed by stacking them together. Given two integers $k_{1}$ and $k_{2}$ with $k_{1}<k_{2}$ , $I\left[k_{1},k_{2}\right]=\left\{k_{1},k_{1}+1,\ldots ,k_{2}\right\}$ . For a vector $x\in R^{n}$ , its norm is defined as $|x|≔{\left(x^{T}x\right)}^{1/2}$ . For a square matrix A , $\lambda_{i}\left(A\right)$ denotes its i -th eigenvalue, while $\lambda_{\text{min}}\left(A\right)$ and $\lambda_{\text{max}}\left(A\right)$ represent its minimum and maximum eigenvalues, respectively.

A directed graph G=(V,E) comprises nodes in the set V={1,2,…,N} and edges in E⊆V×V . An edge from node i to node j is represented as (i,j) , with i as the parent node and j as the child node. Node i is also termed a neighbor of node j . $N_{i}$ is considered as the subset of V consisting of the neighbors of node i . A sequence of edges in G , $\left(i_{1},i_{2}\right),\left(i_{2},i_{3}\right),\ldots ,\left(i_{k},i_{k+1}\right)$ , is called a path from node $i_{1}$ to node $i_{k+1}$ . Node $i_{k+1}$ is reachable from node $i_{1}$ . A directed tree is a graph where each node, except for a root node, has exactly one parent. The root node is reachable from all other nodes. A directed graph G contains a directed spanning tree if at least one node can reach all other nodes. The weighted adjacency matrix of G is a non-negative matrix $A=\left[a_{ij}\right]\in R^{N\times N}$ , where $a_{ii}=0$ and $a_{ij}>0\;\Rightarrow \;\left(j,i\right)\in E$ . The Laplacian of G is denoted as $L=\left[l_{ij}\right]\in R^{N\times N}$ , where $l_{ii}=\sum_{j=1}^{N}a_{ij}$ and $l_{ij}=-a_{ij}$ if i≠j . It is established that L has at least one eigenvalue at the origin, and all nonzero eigenvalues of L have positive real parts. From Ren and Beard (2005), L has one zero eigenvalue and remaining eigenvalues with positive real parts if and only if G has a directed spanning tree.

### 2.2 Radial basis function neural networks (RBFNN)

The RBFNN Networks can be described as $f_{nn}\left(Z\right)=\sum_{i=1}^{N}w_{i}s_{i}\left(Z\right)=W^{T}S\left(Z\right)$ , where $Z\in \Omega_{Z}\subseteq R^{q}$ and $W=w_{1},\ldots ,w_{N}^{T}\in R^{N}$ as input and weight vectors respectively (Park and Sandberg, 1991). N indicates the number of NN nodes, $S\left(Z\right)={\left[s_{1}\left(\left\|Z-\mu_{i}\right\|\right),\ldots ,s_{N}\left(\left\|Z-\mu_{i}\right\|\right)\right]}^{T}$ with $s_{i}\left(\cdot \right)$ is a radial basis function, and $\mu_{i}\left(i=1,\ldots ,N\right)$ is distinct points in the state space. The Gaussian function $s_{i}\left(\left\|Z-\mu_{i}\right\|\right)=\exp\left[-\frac{{\left(Z-\mu_{i}\right)}^{T}\left(Z-\mu_{i}\right)}{\eta_{i}^{2}}\right]$ is generally used for radial basis function, where $\mu_{i}={\left[\mu_{i1},\mu_{i2},\ldots ,\mu_{iN}\right]}^{T}$ is the center and $\eta_{i}$ is the width of the receptive field. The Gaussian function categorized by localized radial basis function s in the sense that $s_{i}\left(\left\|Z-\mu_{i}\right\|\right)\to 0$ as ‖Z‖→∞ . Moreover, for any bounded trajectory Z(t) within the compact set $\Omega_{Z}$ , f(Z) can be approximated using a limited number of neurons located in a local region along the trajectory $f\left(Z\right)=W_{\zeta }^{*}S_{\zeta }\left(Z\right)+\epsilon_{\zeta }$ . ζ denotes the indices of active RBFNN nodes where $|s_{j_{i}}\left(Z\right)|>\iota$ , based on the state Z(t) . $\epsilon_{\zeta }$ is the approximation error, with $\epsilon_{\zeta }=O\left(\epsilon \right)=O\left(\epsilon^{*}\right)$ , $S_{\zeta }\left(Z\right)={\left[s_{j_{1}}\left(Z\right),\ldots ,s_{j_{\zeta }}\left(Z\right)\right]}^{T}\in R^{N_{\zeta }}$ , $W_{\zeta }^{*}={\left[w_{j_{1}}^{*},\ldots ,w_{j_{\zeta }}^{*}\right]}^{T}\in R^{N_{\zeta }}$ , $N_{\zeta }<N_{n}$ , and the integers $j_{i}=j_{1},\ldots ,j_{\zeta }$ are defined by $|s_{j_{i}}\left(Z_{p}\right)|>\iota$ ( ι>0 is a small positive constant) for some $Z_{p}\in Z\left(t\right)$ . This holds if $\|Z\left(t\right)-\xi_{j_{i}}\|<\epsilon$ for t>0 . The following lemma regarding the persistent excitation (PE) condition for RBFNN is recalled from Wang and Hill (2018).

Lemma 1 Consider any continuous recurrent trajectory^1^ $Z\left(t\right):\left[0,\infty \right)\to R^{q}$ . Z(t) remains in a bounded compact set $\Omega_{Z}\subset R^{q}$ . Then for an RBFNN $W^{T}S\left(Z\right)$ with centers placed on a regular lattice (large enough to cover the compact set $\Omega_{Z}$ ), the regressor subvector $S_{\zeta }\left(Z\right)$ consisting of RBFNN with centers located in a small neighborhood of Z(t) is persistently exciting.

### 2.3 Problem statement

A multi-agent system comprising N AUVs with heterogeneous nonlinear uncertain dynamics is considered. The dynamics of each AUV can be expressed as Fossen (1999):

$$\begin{array}{cc} & {\dot{\eta }}_{i}=J_{i}\left(\eta_{i}\right)\nu_{i},\\ & M_{i}{\dot{\nu }}_{i}+C_{i}\left(\nu_{i}\right)\nu_{i}+D_{i}\left(\nu_{i}\right)\nu_{i}+g_{i}\left(\eta_{i}\right)+\Delta_{i}\left(\chi_{i}\right)=\tau_{i}. \end{array}$$ (1)

In this study, the subscript i∈I[1,N] identifies each AUV within the multi-agent system. For every i∈I[1,N] , the vector $\eta_{i}={\left[x_{i},y_{i},\psi_{i}\right]}^{T}\in R^{3}$ represents the i -th AUV’s position coordinates and heading in the global coordinate frame, while $\nu_{i}={\left[u_{i},v_{i},r_{i}\right]}^{T}\in R^{3}$ denotes its linear velocities and angular rate of heading relative to a body-fixed frame. The positive definite inertial matrix $M_{i}=M_{i}^{T}\in S_{3}^{+}$ , Coriolis and centripetal matrix $C_{i}\left(\nu_{i}\right)\in R^{3\times 3}$ , and damping matrix $D_{i}\left(\nu_{i}\right)\in R^{3\times 3}$ characterize the AUV’s dynamic response to motion. The vector $g_{i}\left(\eta_{i}\right)\in R^{3\times 1}$ accounts for the restoring forces and moments due to gravity and buoyancy. The term $\Delta_{i}\left(\chi_{i}\right)\in R^{3\times 1}$ , with $\chi_{i}≔\text{col}\left\{\eta_{i},\nu_{i}\right\}$ , describes the vector of generalized deterministic unmodeled uncertain dynamics for each AUV.

The vector $\tau_{i}\in R^{3}$ represents the control inputs for each AUV. The associated rotation matrix $J_{i}\left(\eta_{i}\right)$ is given by:

$$\begin{array}{c} J_{i}\left(\eta_{i}\right)=\left[\begin{array}{ccc} \cos\left(\psi_{i}\right) & \sin\left(\psi_{i}\right) & 0\\ -\sin\left(\psi_{i}\right) & \cos\left(\psi_{i}\right) & 0\\ 0 & 0 & 1 \end{array}\right], \end{array}$$

Unlike previous work Yuan et al. (2017), which assumed known values for the AUV’s inertia and rotation matrices, this study considers all matrix coefficients, including $C_{i}\left(\nu_{i}\right)$ , $D_{i}\left(\nu_{i}\right)$ , $g_{i}\left(\eta_{i}\right)$ , and $\Delta_{i}\left(\chi_{i}\right)$ , as well as the inertia matrix, as completely unknown. The adaptive estimation process inherently addresses the effects of external forces and disturbances on system dynamics. This eliminates the need for explicit parameter estimation of these forces, as disturbances like water flow, varying currents, or depth variations are directly incorporated into the control input through adaptive estimation. This makes the controller universally applicable to any AUV, regardless of its design, weight, or environmental conditions by addressing both internal and external dynamic variation at the same time.

Internally, it handles unknown parameters such as mass and damping coefficients, as well as unmodeled nonlinear interactions and couplings. Externally, it accounts for unpredictable disturbances, including fluctuating water currents, depth-dependent pressures, and changes in hydrodynamic forces.

By avoiding reliance on predefined models, the proposed approach is robust and adaptable to diverse mission scenarios and unexpected environmental changes, ensuring reliable performance even in highly uncertain conditions.

In the context of leader-following formation tracking control, the following virtual leader dynamics generates the tracking reference signals:

$${\dot{\chi }}_{0}=A_{0}\chi_{0},$$ (2)

with “0” marking the leader node, the leader state $\chi_{0}≔\mathrm{col}\left\{\eta_{0},\nu_{0}\right\}$ with $\eta_{0}\in R^{3}$ and $\nu_{0}\in R^{3}$ , $A_{0}\in R^{6\times 6}$ is a constant matrix available only to the leader’s neighboring AUV agents.

Considering the system dynamics of multiple AUVs (Equation 1) along with the leader dynamics (Equation 2), we establish a non-negative matrix $A=\left[a_{ij}\right]$ , where i,j∈I[0,N] such that for each i∈I[1,N] , $a_{i0}>0$ if and only if agent i has access to the reference signals $\eta_{0}$ and $\nu_{0}$ . All remaining elements of A are arbitrary non-negative values, such that $a_{ii}=0$ for all i . Correspondingly, we establish G=(V,E) as a directed graph derived from A , where V={0,1,…,N} designates node 0 as the leader, and the remaining nodes correspond to the N AUV agents. We proceed under the following assumptions:

Assumption 1 All the eigenvalues of $A_{0}$ in the leader’s dynamics (Equation 2) are located on the imaginary axis.

Assumption 2 The directed graph G contains a directed spanning tree with the node 0 as its root.

Assumption 1 is crucial for ensuring that the leader dynamics produce stable, meaningful reference trajectories for formation control. It ensures that all states of the leader, represented by $\chi_{0}=\text{col}\left\{\eta_{0},\nu_{0}\right\}$ , remain within the bounds of a compact set $\Omega_{0}\subset R^{6}$ for all t≥0 . The trajectory of the system, starting from $\chi_{0}\left(0\right)$ and denoted by $\phi_{0}\left(\chi_{0}\left(0\right)\right)$ , generates periodic signal. This periodicity is essential for maintaining the Persistent Excitation (PE) condition, which is pivotal for achieving parameter convergence in Distributed Adaptive (DA) control systems. Modifications to the eigenvalue constraints on $A_{0}$ mentioned in Assumption 1 may be considered when focusing primarily on formation tracking control performance, as discussed later.

Additionally, Assumption 2 reveals key insights into the structure of the Laplacian matrix L of the network graph G . Let Ψ be an N×N non-negative diagonal matrix where each i -th diagonal element is $a_{i0}$ for i∈I[1,N] . The Laplacian L is formulated as:

$$L=\left[\begin{array}{cc} \overset{N}{\underset{j=1}{\sum }}a_{0j} & -\left[a_{01},\ldots ,a_{0N}\right]\\ -\Psi 1_{N} & H \end{array}\right],$$

where $a_{0j}>0$ if (j,0)∈E and $a_{0j}=0$ otherwise. This results in $H1_{N}=\Psi 1_{N}$ since $L1_{N+1}=0$ . As cited in Su and Huang (2011), all nonzero eigenvalues of H , if present, exhibit positive real parts, confirming H as nonsingular under Assumption 2.

Problem 1 In the context of a multi-AUV system (Equation 1) integrated with virtual leader dynamics (Equation 2) and operating within a directed network topology G , the aim is to develop a distributed NN learning control protocol that leverages only local information. The specific goals are twofold:

1) Formation Control: Each of the N AUV agents will adhere to a predetermined formation pattern relative to the leader, maintaining a specified distance from the leader’s position $\eta_{0}$ .
2) Decentralized Learning: The nonlinear uncertain dynamics of each AUV will be identified and learned autonomously during the formation control process. The insights gained from this learning process will be utilized to enhance the stability and performance of the formation control system.

Remark 1 The leader dynamics described in Equation 2 are designed as a neutrally stable LTI system. This design choice facilitates the generation of sinusoidal reference trajectories at various frequencies which is essential for effective formation tracking control. This approach to leader dynamics is prevalent in the literature on multiagent leader-following distributed control systems like Yuan (2017) and Jandaghi et al. (2024).

Remark 2 It is important to emphasize that the formulation assumes formation control is required only within the horizontal plane, suitable for AUVs operating at a constant depth, and that the vertical dynamics of the 6 degrees of freedom (DOF) AUV system, as detailed in Prestero (2001), are entirely decoupled from the horizontal dynamics.

**FIGURE 1:**
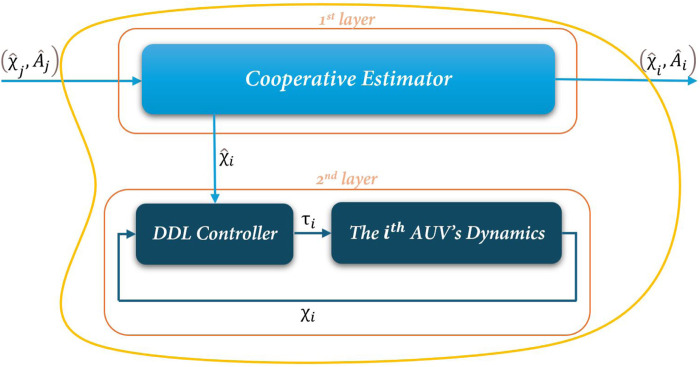
Proposed two-layer distributed controller architecture for each AUVs.

As shown in Figure 1, a two-layer hierarchical design approach is proposed to address the aforementioned challenges. The first layer, the Cooperative Estimator, enables information exchange among neighboring agents. The second layer, known as the Decentralized Deterministic Learning (DDL) controller, processes only local data from each individual AUV. The development and formulation of the first layer are discussed in detail in Section 3.1, while the DDL control strategy, along with its corresponding controller design and analysis, is provided in Section 3.2.

## 3 Two-layer distributed controller architecture

### 3.1 First layer: cooperative estimator

In the context of leader-following formation control, not all AUV agents may have direct access to the leader’s information, including tracking reference signals $\left(\chi_{0}\right)$ and the system matrix $\left(A_{0}\right)$ . This necessitates collaborative interactions among the AUV agents to estimate the leader’s information effectively. Drawing on principles from multiagent consensus and graph theories Ren and Beard (2008), we propose to develop a distributed adaptive observer for the AUV systems as:

$${\dot{\hat{\chi }}}_{i}\left(t\right)={\hat{A}}_{i}\left(t\right){\hat{\chi }}_{i}\left(t\right)+\beta_{i1}\overset{N}{\underset{j=1}{\sum }}a_{ij}\left({\hat{\chi }}_{j}\left(t\right)-{\hat{\chi }}_{i}\left(t\right)\right),\;\forall i\in I\left[1,N\right].$$ (3)

The observer states for each i -th AUV, denoted by ${\hat{\chi }}_{i}={\left[{\hat{\eta }}_{i},{\hat{\nu }}_{i}\right]}^{T}\in R^{6}$ , aim to estimate the leader’s state, $\chi_{0}={\left[\eta_{0},\nu_{0}\right]}^{T}\in R^{6}$ . As t→∞ , these estimates are expected to converge, such that ${\hat{\eta }}_{i}$ approaches $\eta_{0}$ and ${\hat{\nu }}_{i}$ approaches $\nu_{0}$ , representing the leader’s position and velocity, respectively. Equation 3 accounts for the communication graph by including the adjacency matrix information through the term $a_{ij}$ . Note that ${\hat{A}}_{i}\left(t\right)\;\in R^{6\times 6}$ represents an estimate computed by agent i of the leader’s matrix dynamics $A_{0}\left(t\right)\;\in R^{6\times 6}$ which is also not available to the agents. Therefore, each agent estimates such matrix using a cooperative adaptation law:

$${\dot{\hat{A}}}_{i}\left(t\right)=\beta_{i2}\overset{N}{\underset{j=1}{\sum }}a_{ij}\left({\hat{A}}_{j}\left(t\right)-{\hat{A}}_{i}\left(t\right)\right),\;\forall i\in I\left[1,N\right],$$ (4)

which borrowed from Ren and Beard (2008) as well. The constants $\beta_{i1}$ and $\beta_{i2}$ are all positive numbers and are subject to design.

Remark 3 Each AUV agent in the group is equipped with an observer configured as specified in Equations 3, 4, comprising two state variables, $\chi_{i}$ and $A_{i}$ . For each i∈I[1,N] , ${\hat{\chi }}_{i}$ estimates the virtual leader’s state $\chi_{0}$ , while ${\hat{A}}_{i}$ estimates the leader’s system matrix $A_{0}$ . The real-time data necessary for operating the i -th observer includes: (1) the estimated state ${\hat{\chi }}_{i}$ and matrix $\hat{A_{i}}$ , obtained from the i -th AUV itself, and (2) the estimated states ${\hat{\chi }}_{j}$ and matrices $\hat{A_{j}}$ for all $j\in N_{i}$ , obtains from the j -th AUV’s neighbors. Note that in Equations 3, 4, if $j\notin N_{i}$ , then $a_{ij}=0$ , indicating that the i -th observer does not utilize information from the j -th AUV agent. This configuration ensures that the proposed distributed observer can be implemented in each local AUV agent using only locally estimated data from the agent itself and its immediate neighbors, without the need for global information such as the size of the AUV group or the network interconnection topology.

To verify the convergence properties, we need to compute the error dynamics. Now we define the estimation error for the state and the system matrix for agent i as ${\tilde{\chi }}_{i}={\hat{\chi }}_{i}-\chi_{0}$ and ${\tilde{A}}_{i}={\hat{A}}_{i}-A_{0}$ , and then we derive the error dynamics:

$$\begin{array}{ll} {\dot{\tilde{\chi }}}_{i}\,\left(t\right) & ={\hat{A}}_{i}\,\left(t\right)\,{\hat{\chi }}_{i}\,\left(t\right)-A_{0}\,\chi_{0}\,\left(t\right)+\beta_{i1}\sum_{j=1}^{N}a_{ij}\,\left({\hat{\chi }}_{j}\,\left(t\right)-{\hat{\chi }}_{i}\,\left(t\right)\right)\\ & ={\hat{A}}_{i}\,\left(t\right)\,{\hat{\chi }}_{i}\,\left(t\right)-A_{0}\,{\hat{\chi }}_{i}\,\left(t\right)+A_{0}\,{\hat{\chi }}_{i}\,\left(t\right)-A_{0}\,\chi_{0}\,\left(t\right)\\ & \;+\beta_{i1}\sum_{j=1}^{N}a_{ij}\,\left({\hat{\chi }}_{j}\,\left(t\right)-\chi_{0}\,\left(t\right)+\chi_{0}\,\left(t\right)-{\hat{\chi }}_{i}\,\left(t\right)\right)\\ & =A_{0}\,{\tilde{\chi }}_{i}\,\left(t\right)+{\tilde{A}}_{i}\,\left(t\right)\,{\tilde{\chi }}_{i}\,\left(t\right)+{\tilde{A}}_{i}\,\left(t\right)\,\chi_{0}\,\left(t\right)+\beta_{i1}\sum_{j=1}^{N}a_{ij}\,\left({\tilde{\chi }}_{j}\,\left(t\right)-{\tilde{\chi }}_{i}\,\left(t\right)\right)\\ {\dot{\tilde{A}}}_{i}\,\left(t\right) & =\beta_{i2}\sum_{j=1}^{N}a_{ij}\,\left({\tilde{A}}_{j}\,\left(t\right)-{\tilde{A}}_{i}\,\left(t\right)\right),\;\forall i\in I\left[1,N\right]. \end{array}$$

Define the collective error states and adaptation matrices: $\tilde{\chi }=\text{col}\left\{{\tilde{\chi }}_{1},\ldots ,{\tilde{\chi }}_{N}\right\}$ for the state errors, $\tilde{A}=\text{col}\left\{{\tilde{A}}_{1},\ldots ,{\tilde{A}}_{N}\right\}$ for the adaptive parameter errors, ${\tilde{A}}_{b}=\text{diag}\left\{{\tilde{A}}_{1},\ldots ,{\tilde{A}}_{N}\right\}$ representing the block diagonal of adaptive parameters, $B_{\beta_{1}}=\text{diag}\left\{\beta_{11},\ldots ,\beta_{N1}\right\}$ and $B_{\beta_{2}}=\text{diag}\left\{\beta_{12},\ldots ,\beta_{N2}\right\}$ for the diagonal matrices of design constants. With these definitions, the network-wide error dynamics can be expressed as:

$$\begin{array}{ll} \dot{\tilde{\chi }}\,\left(t\right) & =\left(\left(I_{N}⊗A_{0}\right)-B_{\beta_{1}}\,\left(H⊗I_{6}\right)\right)\,\tilde{\chi }\,\left(t\right)+\left({\tilde{A}}_{b}\,\left(t\right)⊗I_{6}\right)\,\tilde{\chi }\,\left(t\right)+{\tilde{A}}_{b}\,\left(t\right)\,\left(1_{N}⊗\chi_{0}\,\left(t\right)\right),\\ \dot{\tilde{A}}\,\left(t\right) & =-B_{\beta_{2}}\,\left(H⊗I_{6}\right)\,\tilde{A}\,\left(t\right). \end{array}$$ (5)

Theorem 1 Consider the error system Equation 5. Under Assumptions 1, 2, and given that $\beta_{1},\beta_{2}>0$ , it follows that for all i∈I[1,N] and for any initial conditions $\chi_{0}\left(0\right),\chi_{i}\left(0\right),A_{i}\left(0\right)$ , the error dynamics of the adaptive parameters and the states will converge to zero exponentially. Specifically, $\lim_{t\to \infty }{\tilde{A}}_{i}\left(t\right)=0$ and $\lim_{t\to \infty }{\tilde{\chi }}_{i}\left(t\right)=0$ .

This convergence is facilitated by the independent adaptation of each agent’s parameters within their respective error dynamics, represented by the block diagonal structure of ${\tilde{A}}_{b}$ and control gains $B_{\beta_{1}}$ and $B_{\beta_{2}}$ . These matrices ensure that each agent’s parameter updates are governed by local interactions and error feedback, consistent with the decentralized control framework.

Proof: We begin by examining the estimation error dynamics for $\tilde{A}$ as presented in Equation 5. This can be rewritten in the vector form:

$${\dot{\vec{\tilde{A}}}}_{0}\left(t\right)=-\beta_{2}\left(I_{6}⊗\left(H⊗I_{6}\right)\right){\vec{\tilde{A}}}_{0}\left(t\right).$$ (6)

Under Assumption 2, all eigenvalues of H possess positive real parts according to Su and Huang (2011). Consequently, for any positive $\beta_{2}>0$ , the matrix $-\beta_{2}\left(I_{6}⊗\left(H⊗I_{6}\right)\right)$ is guaranteed to be Hurwitz, Which implies the exponential stability of system (Equation 6). Hence, it follows that $\lim_{t\to \infty }{\vec{\tilde{A}}}_{0}\left(t\right)=0$ exponentially, leading to $\lim_{t\to \infty }{\tilde{A}}_{i0}\left(t\right)=0$ exponentially for all i∈I[1,N] . Now, we analyze the error dynamics for $\chi_{0}$ in Equation 5. Based on the previous discussions, We have $\lim_{t\to \infty }{\tilde{A}}_{b}\left(t\right)=0$ exponentially, and the term ${\tilde{A}}_{b}\left(t\right)\left(1_{N}⊗\chi_{0}\left(t\right)\right)$ will similarly decay to zero exponentially. Based on Cai et al. (2015), if the system defined by

$${\dot{\tilde{\chi }}}_{0}\left(t\right)=\left(\left(I_{N}⊗A_{0}\right)-\beta_{1}\left(H⊗I_{6}\right)\right){\tilde{\chi }}_{0}\left(t\right).$$ (7)

is exponentially stable, then $\lim_{t\to \infty }\chi_{0}\left(t\right)=0$ exponentially. With Assumption 1, knowing that all eigenvalues of $A_{0}$ have zero real parts, and since H as nonsingular with all eigenvalues in the right-half plane, system (Equation 7) is exponentially stable for any positive $\beta_{1}>0$ . Consequently, this ensures that $\lim_{t\to \infty }\chi_{0}\left(t\right)=0$ , i.e., $\lim_{t\to \infty }\chi_{i0}\left(t\right)=0$ exponentially for all i∈I[1,N] .

Now, each individual agent can accurately estimate both the state and the system matrix of the leader through cooperative observer estimation Equations 3, 4. This information will be utilized in the DDL controller design for each agent’s second layer, which will be discussed in the following subsection.

### 3.2 Second layer: decentralized deterministic learning controller

To fulfill the overall formation learning control objectives, in this section, we develop the DDL control law for the multi-AUV system defined in Equation 1. We use $d_{i}^{*}$ to denote the desired distance between the position of the i -th AUV agent $\eta_{i}$ and the virtual leader’s position $\eta_{0}$ . Then, the formation control problem is framed as a position tracking control task, where each local AUV agent’s position $\eta_{i}$ is required to track the reference signal $\eta_{d,i}≔\eta_{0}+d_{i}^{*}$ . Besides, due to the inaccessibility of the leader’s state information $\chi_{0}$ for all AUV agents, the tracking reference signal ${\hat{\eta }}_{d,i}≔{\hat{\eta }}_{0,i}+d_{i}^{*}$ is employed instead of the reference signal $\eta_{d,i}$ . As established in Theorem 1, ${\hat{\eta }}_{d,i}$ is autonomously generated by each local agent and will exponentially converge to $\eta_{d,i}$ . This ensures that the DDL controller is feasible and the formation control objectives are achievable for all i∈I[1,N] using ${\hat{\eta }}_{d,i}$ .

To design the DDL control law that addresses the formation tracking control and the precise learning of the AUVs’ complete nonlinear uncertain dynamics at the same time, we will integrate renowned backstepping adaptive control design method outlined in Krstic et al. (1995) along with techniques from Wang and Hill (2018) and Yuan et al. (2017) for deterministic learning using RBFNN. Specifically, for the i -th AUV agent described in system (Equation 1), we define the position tracking error as $z_{1,i}=\eta_{i}-{\hat{\eta }}_{d,i}$ for all i∈I[1,N] . Considering $J_{i}\left(\eta_{i}\right)J_{i}^{T}\left(\eta_{i}\right)=I$ for all i∈I[1,N] , we proceed to:

$${\dot{z}}_{1,i}=J_{i}\left(\eta_{i}\right)\nu_{i}-{\dot{\hat{\eta }}}_{i},\;\forall i\in I\left[1,N\right].$$ (8)

To frame the problem in a more tractable way, we assume $\nu_{i}$ as a virtual control input and $\alpha_{i}$ as a desired virtual control input in our control strategy design, and by implementing them in the above system we have:

$$\begin{array}{cc} z_{2,i} & =\nu_{i}-\alpha_{i},\\ \alpha_{i} & =J_{i}^{T}\left(\eta_{i}\right)\left(-K_{1,i}z_{1,i}+{\dot{\hat{\eta }}}_{i}\right),\;\forall i\in I\left[1,N\right]. \end{array}$$ (9)

A positive definite gain matrix $K_{1,i}\in S_{3}^{+}$ is used for tuning the performance. Substituting $\nu_{i}=z_{2,i}+\alpha_{i}$ into Equation 8 yields:

$${\dot{z}}_{1,i}=J_{i}\left(\eta_{i}\right)z_{2,i}-K_{1,i}z_{1,i},\;\forall i\in I\left[1,N\right].$$

Now we derive the first derivatives of the virtual control input and the desired control input as follows:

$$\begin{array}{cc} {\dot{z}}_{2,i} & ={\dot{\nu }}_{i}-{\dot{\alpha }}_{i}\\ & =M_{i}^{-1}\left(-C_{i}\left(\nu_{i}\right)\nu_{i}-D_{i}\left(\nu_{i}\right)\nu_{i}-g_{i}\left(\eta_{i}\right)-\Delta_{i}\left(\chi_{i}\right)+\tau_{i}\right)-{\dot{\alpha }}_{i}, \end{array}$$

$${\dot{\alpha }}_{i}={\dot{J}}_{i}^{T}\,\left(\eta_{i}\right)\,\left(-K_{1,i}\,z_{1,i}+{\dot{\hat{\eta }}}_{i}\right)+J_{i}^{T}\,\left(\eta_{i}\right)\,\left(K_{1,i}\,{\dot{\hat{\eta }}}_{i}-K_{1,i}\,J_{i}\,\left(\eta_{i}\right)\,\nu_{i}+{\ddot{\hat{\eta }}}_{i}\right),\;\forall i\in I\left[1,N\right].$$ (10)

As previously discussed, unlike earlier research that only identified the matrix coefficients $C_{i}\left(\nu_{i}\right)$ , $D_{i}\left(\nu_{i}\right)$ , $g_{i}\left(\eta_{i}\right)$ , and $\Delta_{i}\left(\chi_{i}\right)$ as unknown system nonlinearities while assuming the mass matrix $M_{i}$ to be known, this work advances significantly by also considering $M_{i}$ as unknown. Consequently, all system dynamic parameters are treated as completely unknown, making the controller fully independent of the robot’s configuration such as its dimensions, mass, or any appendages and the uncertain environmental conditions it encounters, like depth, water flow, and viscosity. This independence is critical as it ensures that the controller does not rely on predefined assumptions about the dynamics, aligning with the main goal of this research. To address these challenges, we define a unique nonlinear function $F_{i}\left(Z_{i}\right)$ that encapsulates all nonlinear uncertainties as follows:

$$F_{i}\left(Z_{i}\right)=M_{i}{\dot{\alpha }}_{i}+C_{i}\left(\nu_{i}\right)\nu_{i}+D_{i}\left(\nu_{i}\right)\nu_{i}+g_{i}\left(\eta_{i}\right)+\Delta_{i}\left(\chi_{i}\right),$$ (11)

where $F_{i}\left(Z_{i}\right)={\left[f_{1,i}\left(Z_{i}\right),f_{2,i}\left(Z_{i}\right),f_{3,i}\left(Z_{i}\right)\right]}^{T}$ and $Z_{i}=\text{col}\left\{\eta_{i},\nu_{i}\right\}\in \Omega_{Z_{i}}\subset R^{6}$ , with $\Omega_{Z_{i}}$ being a bounded compact set. We then employ the following RBFNN to approximate the model dynamics in (Equation 11) expressed by nonlinear functions $F_{i}\left(Z_{i}\right)$ with $f_{k,i}$ for all i∈I[1,N] and k∈I[1,3] as follows:

$$f_{k,i}\left(Z_{i}\right)=W_{k,i}^{*T}S_{ki}\left(Z_{i}\right)+\epsilon_{k,i}\left(Z_{i}\right),$$ (12)

where $W_{k,i}^{*}$ is the ideal constant NN weights, and $\epsilon_{k,i}\left(Z_{i}\right)$ is the approximation error $\epsilon_{k,i}^{*}>0$ for all i∈I[1,N] and k∈I[1,3] , which satisfies $|\epsilon_{k,i}\left(Z_{i}\right)|\leq \epsilon_{k,i}^{*}$ . This error can be made arbitrarily small given a sufficient number of neurons in the network. A self-adaptation law is designed to estimate the unknown $W_{k,i}^{*}$ online. We aim to estimate $W_{k,i}^{*}$ with ${\hat{W}}_{k,i}$ by constructing the DDL feedback control law as follows:

$$\tau_{i}=-J_{i}^{T}\left(\eta_{i}\right)z_{1,i}-K_{2,i}z_{2,i}+{\hat{W}}_{i}^{T}S_{i}^{F}\left(Z_{i}\right).$$ (13)

$K_{2,i}\in S_{3}^{+}$ is a feedback gain matrix that can be tuned to achieve the desired performance. To approximate the unknown nonlinear function vector $F_{i}\left(Z_{i}\right)$ in (Equation 11) along the trajectory $Z_{i}$ within the compact set $\Omega_{Z_{i}}$ , we use:

$${\hat{W}}_{i}^{T}S_{i}^{F}\left(Z_{i}\right)=\left[\begin{array}{c} {\hat{W}}_{1,i}^{T}S_{1,i}\left(Z_{i}\right)\\ {\hat{W}}_{2,i}^{T}S_{2,i}\left(Z_{i}\right)\\ {\hat{W}}_{3,i}^{T}S_{3,i}\left(Z_{i}\right) \end{array}\right].$$

Then, from Equations 1, 13 we have:

$$M_{i}\,{\dot{\nu }}_{i}+C_{i}\,\left(\nu_{i}\right)\,\nu_{i}+D_{i}\,\left(\nu_{i}\right)\,\nu_{i}+g_{i}\,\left(\eta_{i}\right)+\Delta_{i}\,\left(\chi_{i}\right)=\tau_{i}=-J_{i}^{T}\,\left(\eta_{i}\right)\,z_{1,i}-K_{2,i}\,z_{2,i}+{\hat{W}}_{k,i}^{T}\,S_{k,i}\,\left(Z_{i}\right).$$

By subtracting $W_{k,i}^{*T}S_{k,i}\left(Z_{i}\right)+\epsilon_{k,i}\left(Z_{i}\right)$ from both sides and considering Equations 9, 11, we define ${\tilde{W}}_{k,i}≔{\hat{W}}_{k,i}-W_{k,i}^{*}$ , leading to:

$${\dot{z}}_{2,i}=M_{i}^{-1}\left(-J_{i}^{T}\left(\eta_{i}\right)z_{1,i}-K_{2,i}z_{2,i}+{\hat{W}}_{k,i}^{T}S_{k,i}\left(Z_{i}\right)-\epsilon_{k,i}\left(Z_{i}\right)\right).$$

For updating ${\hat{W}}_{k,i}$ online, a robust self-adaptation law is constructed using the σ -modification technique Ioannou and Sun (1996) as follows:

$${\dot{\hat{W}}}_{k,i}=-\Gamma_{k,i}\left(S_{k,i}\left(Z_{i}\right)z_{2k,i}+\sigma_{ki}{\hat{W}}_{k,i}\right).$$ (14)

where $z_{2,i}={\left[z_{21,i},z_{22,i},z_{23,i}\right]}^{T}$ , $\Gamma_{k,i}=\Gamma_{k,i}^{T}>0$ , and $\sigma_{k,i}>0$ are free parameters to be designed for all i∈I[1,N] and k∈I[1,3] . Integrating Equations 9, 13, 14 yields the following closed-loop system:

$$\left\{\begin{array}{cc} {\dot{z}}_{1,i} & =-K_{1,i}z_{1,i}+J_{i}\left(\eta_{i}\right)z_{2,i},\\ {\dot{z}}_{2,i} & =M_{i}^{-1}\left(-J_{i}^{T}\left(\eta_{i}\right)z_{1,i}-K_{2,i}z_{2,i}+{\hat{W}}_{ki}^{T}S_{ki}\left(Z_{i}\right)-\epsilon_{k,i}\left(Z_{i}\right)\right),\\ {\dot{\tilde{W}}}_{k,i} & =-\Gamma_{k,i}\left(S_{k,i}\left(Z_{i}\right)z_{2,k,i}+\sigma_{k,i}{\hat{W}}_{k,i}\right), \end{array}\right.$$ (15)

where, for all i∈I[1,N] and k∈I[1,3] , ${\tilde{W}}_{i}^{T}S_{i}\left(Z_{i}\right)={\left[{\tilde{W}}_{1,i}^{T}S_{1,i}\left(Z_{i}\right),{\tilde{W}}_{2,i}^{T}S_{2,i}\left(Z_{i}\right),{\tilde{W}}_{3,i}^{T}S_{3,i}\left(Z_{i}\right)\right]}^{T}$ , and $\epsilon_{i}\left(Z_{i}\right)={\left[\epsilon_{1,i}\left(Z_{i}\right),\epsilon_{2,i}\left(Z_{i}\right),\epsilon_{3,i}\left(Z_{i}\right)\right]}^{T}$ .

Remark 4 Unlike the first-layer DA observer design, the second-layer control law is fully decentralized for each local agent. It utilizes only the local agent’s information for feedback control, including $\chi_{i}$ , ${\hat{\chi }}_{i}$ , and $W_{k,i}$ , without involving any information exchange among neighboring AUVs.

The following theorem summarizes the stability and tracking control performance results of the overall system:

Theorem 2 Consider the local closed-loop system (Equation 15). For each i∈I[1,N] , if there exists a sufficiently large compact set $\Omega_{Z_{i}}$ such that $Z_{i}\in \Omega_{Z_{i}}$ for all t≥0 , then for any bounded initial conditions, we have: 1) All signals in the closed-loop system remain uniformly ultimately bounded (UUB). 2) The position tracking error $\eta_{i}-\eta_{d,i}$ converges exponentially to a small neighborhood around zero in finite time $T_{i}>0$ by choosing the design parameters with sufficiently large $\underline{\lambda }\left(K_{1,i}\right)>0$ and $\underline{\lambda }\left(K_{2,i}\right)>2\bar{\lambda }\left(K_{1,i}\right)>0$ , and sufficiently small $\sigma_{k,i}>0$ for all i∈I[1,N] and k∈I[1,3] .

**Proof:** 1) Consider the following Lyapunov function candidate for the closed-loop system (Equation 15):

$$V_{i}=\frac{1}{2}z_{1,i}^{T}z_{1,i}+\frac{1}{2}z_{2,i}^{T}M_{i}z_{2,i}+\frac{1}{2}\overset{3}{\underset{k=1}{\sum }}{\tilde{W}}_{k,i}^{T}\Gamma_{k,i}^{-1}{\tilde{W}}_{k,i}.$$

Evaluating the derivative of $V_{i}$ along the trajectory of Equation 15 for all i∈I[1,N] yields:

$$\begin{array}{cc} {\dot{V}}_{i} & =z_{1,i}^{T}\left(-K_{1,i}z_{1,i}+J_{i}\left(\eta_{i}\right)z_{2,i}\right)\\ & \;+z_{2,i}^{T}\left.\left(-J_{i}^{T}\left(\eta_{i}\right)z_{1,i}-K_{2,i}z_{2,i}+{\tilde{W}}_{k,i}^{T}S_{k,i}\left(Z_{i}\right)-\epsilon_{k,i}\left(Z_{i}\right)\right)\right)\\ & \;-\overset{3}{\underset{k=1}{\sum }}{\tilde{W}}_{k,i}^{T}\left(S_{k,i}\left(Z_{i}\right)z_{2k,i}+\sigma_{k,i}{\hat{W}}_{k,i}\right)\\ & =-z_{1,i}^{T}K_{1,i}z_{1,i}-z_{2,i}^{T}K_{2,i}z_{2,i}-z_{2,i}^{T}\epsilon_{k,i}\left(Z_{i}\right)\\ & \;-\overset{3}{\underset{k=1}{\sum }}\sigma_{k,i}{\tilde{W}}_{k,i}^{T}{\hat{W}}_{k,i},\;\forall i\in I\left[1,N\right]. \end{array}$$

Choose $K_{2,i}=K_{1,i}+K_{22,i}$ such that $K_{1,i},K_{22,i}\in S_{3}^{+}$ . Using the completion of squares, we have:

$$\begin{array}{cc} -\sigma_{k,i}{\tilde{W}}_{k,i}^{T}{\hat{W}}_{k,i} & \leq -\frac{\sigma_{k,i}\|{\tilde{W}}_{k,i}{\|}^{2}}{2}+\frac{\sigma_{k,i}\|W_{k,i}^{*}{\|}^{2}}{2},\\ -z_{2,i}^{T}K_{22,i}z_{2,i}-z_{2,i}^{T}\epsilon_{i}\left(Z_{i}\right) & \leq \frac{\epsilon_{i}^{T}\left(Z_{i}\right)\epsilon_{i}\left(Z_{i}\right)}{4\underline{\lambda }\left(K_{22,i}\right)}\leq \frac{\|\epsilon_{i}^{*}{\|}^{2}}{4\underline{\lambda }\left(K_{22,i}\right)}, \end{array}$$

where $\epsilon_{i}^{*}={\left[\epsilon_{1,i}^{*},\epsilon_{2,i}^{*},\epsilon_{3,i}^{*}\right]}^{T}$ . Then, we obtain:

$$\begin{array}{cc} {\dot{V}}_{i}\leq & -z_{1,i}^{T}K_{1,i}z_{1,i}-z_{2,i}^{T}K_{1,i}z_{2,i}+\frac{\|\epsilon_{i}^{*}{\|}^{2}}{4\underline{\lambda }\left(K_{22,i}\right)}\\ & +\overset{3}{\underset{k=1}{\sum }}\left(-\frac{\sigma_{k,i}\|{\tilde{W}}_{k,i}{\|}^{2}}{2}+\frac{\sigma_{k,i}\|W_{k,i}^{*}{\|}^{2}}{2}\right.. \end{array}$$

It follows that ${\dot{V}}_{i}$ is negative definite whenever:

$$\begin{array}{cc} \left\|z_{1,i}\right\| & >\frac{\left\|\epsilon_{i}^{*}\right\|}{2\sqrt{\underline{\lambda }\left(K_{1,i}\right)\underline{\lambda }\left(K_{22,i}\right)}}+\overset{3}{\underset{k=1}{\sum }}\left(\sqrt{\frac{\sigma_{k,i}}{2\underline{\lambda }\left(K_{1,i}\right)}}\|W_{k,i}^{*}\|\right),\\ \left\|z_{2,i}\right\| & >\frac{\left\|\epsilon_{i}^{*}\right\|}{2\sqrt{\underline{\lambda }\left(K_{1,i}\right)\underline{\lambda }\left(K_{22,i}\right)}}+\overset{3}{\underset{k=1}{\sum }}\left(\sqrt{\frac{\sigma_{k,i}}{2\underline{\lambda }\left(K_{1,i}\right)}}\|W_{k,i}^{*}\|\right),\\ \left\|{\tilde{W}}_{k,i}\right\| & >\frac{\left\|\epsilon_{i}^{*}\right\|}{2\sqrt{\sigma_{k,i}\underline{\lambda }\left(K_{22,i}\right)}}+\overset{3}{\underset{k=1}{\sum }}\|W_{k,i}^{*}\|:={\tilde{W}}_{k,i}^{*}. \end{array}$$

For all i∈I[1,N] , ∃k∈I[1,3] . This leads to the Uniformly Ultimately Bounded (UUF) behavior of the signals $z_{1,i}$ , $z_{2,i}$ , and ${\tilde{W}}_{k,i}$ for all i∈I[1,N] and k∈I[1,3] . As a result, it can be easily verified that since $\eta_{d}i=\eta_{i}+d_{i}^{*}$ with $\eta_{i}$ bounded (according to Theorem 1 and Assumption 1), $\eta_{i}=z_{1,i}+\eta_{i}$ is bounded for all i∈I[1,N] . Similarly, the boundedness of $\nu_{i}=z_{2,i}+\alpha_{i}$ can be confirmed by the fact that $\alpha_{i}$ in Equation 9 is bounded. In addition, $W_{k,i}={\tilde{W}}_{k,i}+W_{k,i}^{*}$ is also bounded for all i∈I[1,N] and k∈I[1,3] because of the boundedness of ${\tilde{W}}_{k,i}$ and $W_{k,i}^{*}$ . Moreover, in light of Equation 10, ${\dot{\alpha }}_{i}$ is bounded as all the terms on the right-hand side of Equation 10 are bounded. This leads to the boundedness of the control signal $\tau_{i}$ in Equation 13 since the Gaussian function vector $S_{i}^{F}\left(Z_{i}\right)$ is guaranteed to be bounded for any $Z_{i}$ . As such, all the signals in the closed-loop system remain UUB, which completes the proof of the first part.

2) For the second part, it will be shown that $\eta_{i}$ will converge arbitrarily close to $\eta_{di}$ in some finite time $T_{i}>0$ for all i∈I[1,N] . To this end, we consider the following Lyapunov function candidate for the dynamics of $z_{1,i}$ and $z_{2,i}$ in Equation 15:

$$V_{z,i}=\frac{1}{2}z_{1,i}^{T}z_{1,i}+\frac{1}{2}z_{2,i}^{T}M_{i}z_{2,i},\;\forall i\in I\left[1,N\right].$$ (16)

The derivative of $V_{z,i}$ is:

$$\begin{array}{ll} {\dot{V}}_{z,i} & =z_{1,i}^{T}\,\left(-K_{1,i}\,z_{1,i}+J_{i}\,\left(\eta_{i}\right)\,z_{2,i}\right)+z_{2,i}^{T}\,\left(-J_{i}^{T}\,\left(\eta_{i}\right)\,z_{1,i}-K_{2,i}\,z_{2,i}+{\tilde{W}}_{k,i}^{T}\,S_{k,i}\,\left(Z_{i}\right)-\epsilon_{i}\,\left(Z_{i}\right)\right)\\ & =-z_{1,i}^{T}\,K_{1,i}\,z_{1,i}-z_{2,i}^{T}\,K_{2,i}\,z_{2,i}+z_{2,i}^{T}\,{\tilde{W}}_{k,i}^{T}\,S_{k,i}\,\left(Z_{i}\right)-z_{2,i}^{T}\,\epsilon_{k,i}\,\left(Z_{i}\right),\;\forall i\in I\left[1,N\right]. \end{array}$$

Similar to the proof of part one, we let $K_{2,i}=K_{1,i}+2K_{22,i}$ with $K_{1,i},K_{22,i}\in S_{3}^{+}$ . According to Wang and Hill (2018), the Gaussian RBFNN regressor $S_{i}^{F}\left(Z_{i}\right)$ is bounded by $\|S_{i}^{F}\left(Z_{i}\right)\|\leq s_{i}^{*}$ for any $Z_{i}$ and for all i∈I[1,N] with some positive number $s_{i}^{*}>0$ . Through completion of squares, we have:

$$\begin{array}{cc} -z_{2,i}^{T}K_{22,i}z_{2,i}+z_{2,i}^{T}{\tilde{W}}_{i}^{T}S_{i}^{F}\left(Z_{i}\right) & \leq \frac{\|{\tilde{W}}_{i}^{*}{\|}^{2}s_{i}^{*2}}{4\underline{\lambda }\left(K_{22,i}\right)},\\ -z_{2,i}^{T}K_{22,i}z_{2,i}-z_{2,i}^{T}\epsilon_{i}\left(Z_{i}\right) & \leq \frac{\|\epsilon_{i}^{*}{\|}^{2}}{4\underline{\lambda }\left(K_{22,i}\right)}. \end{array}$$

Also ${\tilde{W}}_{i}^{*}={\left[{\tilde{W}}_{1,i}^{*},{\tilde{W}}_{2,i}^{*},{\tilde{W}}_{3,i}^{*}\right]}^{T}$ . This leads to:

$$\begin{array}{cc} \;{\dot{V}}_{z,i} & \leq -z_{1,i}^{T}K_{1,i}z_{1,i}-z_{2,i}^{T}K_{1,i}z_{2,i}+\delta_{i},\\ & \leq -2\underline{\lambda }\left(K_{1,i}\right)\left(\frac{1}{2}z_{1,i}^{T}z_{1,i}+\frac{1}{2\bar{\lambda }\left(M_{i}\right)}z_{2,i}^{T}M_{i}z_{2,i}\right)+\delta_{i},\\ & \leq -\rho_{i}V_{z,i}+\delta_{i},\;\forall i\in I\left[1,N\right], \end{array}$$ (17)

where $\rho_{i}=\min\left\{2\underline{\lambda }\left(K_{1,i}\right),2\underline{\lambda }\left(K_{1,i}\right)/\bar{\lambda }\left(M_{i}\right)\right\}$ and $\delta_{i}=\left(\|{\tilde{W}}_{i}^{*}{\|}^{2}s_{i}^{*2}/4\underline{\lambda }\left(K_{22,i}\right)\right)+\left(\|\epsilon_{i}^{*}{\|}^{2}/4\underline{\lambda }\left(K_{22,i}\right)\right)$ , ∀i∈I[1,N] . Solving the inequality Equation 17 yields:

$$0\leq V_{z,i}\left(t\right)\leq V_{z,i}\left(0\right)\exp\left(-\rho_{i}t\right)+\frac{\delta_{i}}{\rho_{i}},$$

which together with Equation 16 implies that:

$$\min\left\{1,\underline{\lambda }\,\left(M_{i}\right)\right\}\frac{1}{2}\,\left(\|z_{1,i}\,{\|}^{2}+\|z_{2,i}\,{\|}^{2}\right)\leq V_{z,i}\,\left(0\right)\,\exp\left(-\rho_{i}\,t\right)+\frac{\delta_{i}}{\rho_{i}},\;\forall t\geq 0,\;i\in I\left[1,N\right],$$

also

$$\|z_{1,i}\,{\|}^{2}+\|z_{2,i}\,{\|}^{2}\leq \frac{2}{\min\left\{1,\underline{\lambda }\left(M_{i}\right)\right\}}\,V_{z,i}\,\left(0\right)\,\exp\left(-\rho_{i}\,t\right)+\frac{2\delta_{i}}{\rho_{i}\min\left\{1,\underline{\lambda }\left(M_{i}\right)\right\}}.$$

Consequently, it is straightforward that given ${\bar{\delta }}_{i}>\sqrt{2\delta_{i}/\rho_{i}\min\left\{1,\underline{\lambda }\left(M_{i}\right)\right\}}$ , there exists a finite time $T_{i}>0$ for all i∈I[1,N] such that for all $t\geq T_{i}$ , both $z_{1,i}$ and $z_{2,i}$ satisfy $\|z_{1,i}\left(t\right)\|\leq {\bar{\delta }}_{i}$ and $\|z_{2,i}\left(t\right)\|\leq {\bar{\delta }}_{i}\;\forall i\in I\left[1,N\right]$ , where ${\bar{\delta }}_{i}$ can be made arbitrarily small by choosing sufficiently large $\underline{\lambda }\left(K_{1,i}\right)>0$ and $\underline{\lambda }\left(K_{2,i}\right)>2\bar{\lambda }\left(K_{1,i}\right)>0$ for all i∈I[1,N] . This ends the proof.

By integrating the outcomes of Theorems 1, 2, the following theorem is established, which can be presented without additional proof:

Theorem 3 By Considering the multi-AUV system (Equation 1) and the virtual leader dynamics (Equation 2) with the network communication topology G and under Assumptions 1 and 2, the objective 1 of Problem 1 (i.e., $\eta_{i}$ converges to $\eta_{0}+d_{i}^{*}$ exponentially for all i∈I[1,N] ) can be achieved by using the cooperative observer Equations 3, 4 and the DDL control law Equations 13, 14 with all the design parameters satisfying the requirements in Theorems 1 and 2, respectively.

Remark 5 With the proposed two-layer formation learning control architecture, inter-agent information exchange occurs solely in the first-layer DA observation. Only the observer’s estimated information, and not the physical plant state information, needs to be shared among neighboring agents. Additionally, since no global information is required for the design of each local AUV control system, the proposed formation learning control protocol can be designed and implemented in a fully distributed manner.

Remark 6 It is important to note that the eigenvalue constraints on $A_{0}$ in Assumption 1 are not needed for cooperative observer estimation (as detailed in the Section 3 or for achieving formation tracking control performance (as discussed in this section). This indicates that formation tracking control can be attained for general reference trajectories, including both periodic paths and straight lines, provided they are bounded. However, these constraints will become necessary in the next section to ensure the accurate learning capability of the proposed method.

## 4 Accurate learning from formation control

It is necessary to demonstrate the convergence of the RBFNN weights in Equations 13, 14 to their optimal values for accurate learning and identification. The main result of this section is summarized in the following theorem.

Theorem 4 Consider the local closed-loop system (Equation 15) with Assumptions 1, 2. For each i∈I[1,N] , if there exists a sufficiently large compact set $\Omega_{Z_{i}}$ such that $Z_{i}\in \Omega_{Z_{i}}$ for all t≥0 , then for any bounded initial conditions and $W_{k,i}\left(0\right)=0\;\forall i\in I\left[1,N\right],k\in I\left[1,3\right]$ , the local estimated neural weights $W_{\zeta ,k,i}$ converge to small neighborhoods of their optimal values $W_{\zeta ,k,i}^{*}$ along the periodic reference tracking orbit $\phi_{\zeta ,i}\left(Z_{i}\left(t\right)\right){|}_{t\geq T_{i}}$ (denoting the orbit of the NN input signal $Z_{i}\left(t\right)$ starting from time $T_{i}$ ). This leads to locally accurate approximations of the nonlinear uncertain dynamics $f_{k,i}\left(Z_{i}\right)\;\forall k\in I\left[1,3\right]$ in Equation 11 being obtained by $W_{k,i}^{T}S_{k,i}\left(Z_{i}\right)$ , as well as by ${\bar{W}}_{k,i}^{T}S_{k,i}\left(Z_{i}\right)$ , where ∀i∈I[1,N],k∈I[1,3] . Also, ζ denotes the subset of neurons (or nodes) that are active when the system state Z(t) is within a specific neighborhood of the state space.

$${\bar{W}}_{k,i}={mean}_{t\in \left[t_{a,i},t_{b,i}\right]}{\hat{W}}_{k,i}\left(t\right),$$ (18)

where $\left[t_{a,i},t_{b,i}\right]$ $\left(t_{b,i}>t_{a,i}>T_{i}\right)$ represents a time segment after the transient process.

Proof: From Theorem 3, we have shown that for all i∈I[1,N] , $\eta_{i}$ will closely track the periodic signal $\eta_{d,i}=\eta_{0}+d_{i}^{*}$ in finite time $T_{i}$ . In addition, (Equation 9) implies that $\nu_{i}$ will also closely track the signal $J_{i}^{T}\left(\eta_{i}\right){\dot{\eta }}_{0}^{i}$ since both $z_{1,i}$ and $z_{2,i}$ will converge to a small neighborhood around zero according to Theorem 2. Moreover, since ${\dot{\eta }}_{0}^{i}$ will converge to ${\dot{\eta }}_{0}$ according to Theorem 1, and $J_{i}\left(\eta_{i}\right)$ is a bounded rotation matrix, $\nu_{i}$ will also be a periodic signal after finite time $T_{i}$ , because ${\dot{\eta }}_{0}$ is periodic under Assumption 1. Consequently, since the RBFNN input $Z_{i}\left(t\right)=col\left\{\eta_{i},\nu_{i}\right\}$ becomes a periodic signal for all $t\leq T_{i}$ , the PE condition of some internal closed-loop signals, i.e., the RBFNN regression subvector $S_{\zeta ,k,i}\left(Z_{i}\right)$ $\left(\forall t\geq T_{i}\right)$ , is satisfied according to Lemma 1. As mentioned in Section 2.2, ζ represents the subset of RBFNN nodes and weights that are specifically utilized along the recurrent trajectory of the system state $Z_{i}\left(t\right)$ . This subset focuses on the active neural components required for approximating the system’s nonlinear dynamics locally, ensuring that the learning and adaptation processes are efficient and accurate within the compact region where the trajectory resides. It should be noted that the periodicity of $Z_{i}\left(t\right)$ leads to the PE of the regression subvector $S_{\zeta ,k,i}\left(Z_{i}\right)$ , but not necessarily the PE of the whole regression vector $S_{k,i}\left(Z_{i}\right)$ . Thus, we term this as a partial PE condition, and we will show the convergence of the associated local estimated neural weights $W_{\zeta ,k,i}\to W_{\zeta ,k,i}^{*}$ , rather than $W_{k,i}\to W_{k,i}^{*}$ .

Thus, to prove accurate convergence of local neural weights $W_{\zeta ,k,i}$ associated with the regression subvector $S_{\zeta ,k,i}\left(Z_{i}\right)$ under the satisfaction of the partial PE condition, we first rewrite the closed-loop dynamics of $z_{1,i}$ and $z_{2,i}$ along the periodic tracking orbit $\phi_{\zeta ,i}\left(Z_{i}\left(t\right)\right){|}_{t\geq T_{i}}$ by using the localization property of the Gaussian RBFNN:

$$\begin{array}{ll} {\dot{z}}_{1,i} & =-K_{1,i}\,z_{1,i}+J_{i}\,\left(\eta_{i}\right)\,z_{2,i},\\ {\dot{z}}_{2,i} & =M_{i}^{-1}\,\left(-W_{\zeta ,i}^{*T}\,S_{\zeta ,i}^{F}\,\left(Z_{i}\right)-\epsilon_{\zeta ,i}-J_{i}^{T}\,\left(\eta_{i}\right)\,z_{1,i}-K_{2,i}\,z_{2,i}+{\hat{W}}_{\zeta ,i}^{T}\,S_{\zeta ,i}^{F}\,\left(Z_{i}\right)+{\hat{W}}_{\bar{\zeta },i}^{T}\,S_{\bar{\zeta },i}^{F}\,\left(Z_{i}\right)\right)\\ & =M_{i}^{-1}\,\left(-J_{i}^{T}\,\left(\eta_{i}\right)\,z_{1,i}-K_{2,i}\,z_{2,i}+{\tilde{W}}_{\zeta ,i}^{T}\,S_{\zeta ,i}^{F}\,\left(Z_{i}\right)-\epsilon_{\zeta ,i}^{\prime }\right). \end{array}$$

where $F_{i}\left(Z_{i}\right)=W_{\zeta ,i}^{*T}S_{\zeta ,i}^{F}\left(Z_{i}\right)+\epsilon_{\zeta ,i}$ with $W_{\zeta ,i}^{*T}S_{\zeta ,i}^{F}\left(Z_{i}\right)={\left[W_{\zeta ,1,i}^{*T}S_{\zeta ,1,i}\left(Z_{i}\right),W_{\zeta ,2,i}^{*T}S_{\zeta ,2,i}\left(Z_{i}\right),W_{\zeta ,3,i}^{*T}S_{\zeta ,3,i}\left(Z_{i}\right)\right]}^{T}$ and $\epsilon_{\zeta ,i}={\left[\epsilon_{\zeta ,1,i},\epsilon_{\zeta ,2,i},\epsilon_{\zeta ,3,i}\right]}^{T}$ being the approximation error. Additionally, $W_{\zeta ,i}^{T}S_{\zeta ,i}^{F}\left(Z_{i}\right)+W_{\bar{\zeta },i}^{T}S_{\bar{\zeta },i}^{F}\left(Z_{i}\right)=W_{i}^{T}S_{i}^{F}\left(Z_{i}\right)$ with subscripts ζ and $\bar{\zeta }$ denoting the regions close to and far away from the periodic trajectory $\phi_{\zeta ,i}\left(Z_{i}\left(t\right)\right){|}_{t\geq T_{i}}$ , respectively. According to Wang and Hill (2018), $\left\|W_{\bar{\zeta },i}^{T}S_{\bar{\zeta },i}^{F}\left(Z_{i}\right)\right\|$ is small, and the NN local approximation error $\epsilon_{\zeta ,i}^{\prime }=\epsilon_{\zeta ,i}-W_{\bar{\zeta },i}^{T}S_{\bar{\zeta },i}^{F}\left(Z_{i}\right)$ with $\|\epsilon_{\zeta ,i}^{\prime }\|=O\left(\left\|\epsilon_{\zeta ,i}\right\|\right)$ is also a small number. Thus, the overall closed-loop adaptive learning system can be described by:

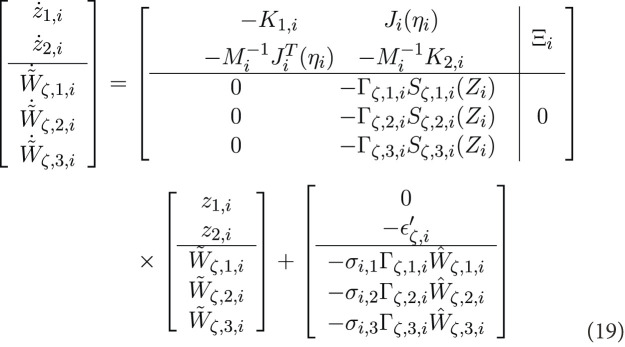

and

$$\begin{array}{cc} \left[\begin{array}{c} {\dot{\tilde{W}}}_{\bar{\zeta },i,1}\\ {\dot{\tilde{W}}}_{\bar{\zeta },i,2}\\ {\dot{\tilde{W}}}_{\bar{\zeta },i,3} \end{array}\right] & =\left[\begin{array}{c} -\Gamma_{\bar{\zeta },1,i}\left(S_{\bar{\zeta },1,i}\left(Z_{i}\right)z_{2,i}+\sigma_{i,1}{\hat{W}}_{\bar{\zeta },1,i}\right)\\ -\Gamma_{\bar{\zeta },2,i}\left(S_{\bar{\zeta },2,i}\left(Z_{i}\right)z_{2,i}+\sigma_{i,2}{\hat{W}}_{\bar{\zeta },2,i}\right)\\ -\Gamma_{\bar{\zeta },3,i}\left(S_{\bar{\zeta },3,i}\left(Z_{i}\right)z_{2,i}+\sigma_{i,3}{\hat{W}}_{\bar{\zeta },3,i}\right) \end{array}\right], \end{array}$$

where

$$\begin{array}{cc} \Xi_{i} & =\left[\begin{array}{c} 0\\ M_{i}^{-1}\left[\begin{array}{ccc} S_{\zeta ,1,i}^{T}\left(Z_{i}\right) & 0 & 0\\ 0 & S_{\zeta ,2,i}^{T}\left(Z_{i}\right) & 0\\ 0 & 0 & S_{\zeta ,3,i}^{T}\left(Z_{i}\right) \end{array}\right] \end{array}\right], \end{array}$$

for all i∈I[1,N] . The exponential stability property of the nominal part of subsystem (Equation 19) has been well-studied in Wang and Hill (2018), Yuan and Wang (2011), and Yuan and Wang (2012), where it is stated that PE of $S_{\zeta ,k,i}\left(Z_{i}\right)$ will guarantee exponential convergence of $\left(z_{1,i},z_{2,i},{\tilde{W}}_{\zeta ,k,i}\right)=0$ for all i∈I[1,N] and k∈I[1,3] . Based on this, since $\|\epsilon_{\zeta ,i}^{\prime }\|=O\left(\left\|\epsilon_{\zeta ,i}\right\|\right)=O\left(\left\|\epsilon_{i}\right\|\right)$ , and $\sigma_{k,i}\Gamma_{\zeta ,k,i}{\hat{W}}_{\zeta ,k,i}$ can be made small by choosing sufficiently small $\sigma_{k,i}$ for all i∈I[1,N] , k∈I[1,3] , both the state error signals $\left(z_{1,i},z_{2,i}\right)$ and the local parameter error signals ${\tilde{W}}_{\zeta ,k,i}$ (∀i∈I[1,N],k∈I[1,3]) will converge exponentially to small neighborhoods of zero, with the sizes of the neighborhoods determined by the RBFNN ideal approximation error $\epsilon_{i}$ as in Equation 12 and $\sigma_{k,i}\|\Gamma_{\zeta ,k,i}{\hat{W}}_{\zeta ,k,i}\|$ . The convergence of $W_{\zeta ,k,i}\to W_{\zeta ,k,i}^{*}$ implies that along the periodic trajectory $\phi_{\zeta ,i}\left(Z_{i}\left(t\right)\right){|}_{t\geq T_{i}}$ , we have

$$\begin{array}{cc} f_{k,i}\left(Z_{i}\right) & =W_{\zeta ,k,i}^{*T}S_{\zeta ,k,i}\left(Z_{i}\right)+\epsilon_{\zeta ,k,i}\\ & ={\hat{W}}_{\zeta ,k,i}^{T}S_{\zeta ,k,i}\left(Z_{i}\right)-{\tilde{W}}_{\zeta ,k,i}^{T}S_{\zeta ,k,i}\left(Z_{i}\right)+\epsilon_{\zeta ,k,i}\\ & ={\hat{W}}_{\zeta ,k,i}^{T}S_{\zeta ,k,i}\left(Z_{i}\right)+\epsilon_{\zeta_{1},k,i}\\ & ={\bar{W}}_{\zeta ,k,i}^{T}S_{\zeta ,k,i}\left(Z_{i}\right)+\epsilon_{\zeta_{2},k,i}, \end{array}$$

where for all i∈I[1,N] , k∈I[1,3] , $\epsilon_{\zeta_{1},k,i}=\epsilon_{\zeta ,k,i}-{\tilde{W}}_{\zeta ,k,i}^{T}S_{\zeta ,k,i}\left(Z_{i}\right)=O\left(\left\|\epsilon_{\zeta ,i}\right\|\right)$ due to the convergence of ${\tilde{W}}_{\zeta ,k,i}\to 0$ . The last equality is obtained according to the definition of (Equation 18) with ${\bar{W}}_{\zeta ,k,i}$ being the corresponding subvector of ${\bar{W}}_{k,i}$ along the periodic trajectory $\phi_{\zeta ,i}\left(Z_{i}\left(t\right)\right){|}_{t\geq T_{i}}$ , and $\epsilon_{\zeta_{2},k,i}$ being an approximation error using ${\bar{W}}_{\zeta ,k,i}^{T}S_{\zeta ,k,i}\left(Z_{i}\right)$ . Apparently, after the transient process, we will have $\epsilon_{\zeta_{2},k,i}=O\left(\epsilon_{\zeta_{1},k,i}\right)$ , ∀i∈I[1,N] , k∈I[1,3] . Conversely, for the neurons whose centers are distant from the trajectory $\phi_{\zeta ,i}\left(Z_{i}\left(t\right)\right){|}_{t\geq T_{i}}$ , the values of $\left\|S_{\bar{\zeta },k,i}\left(Z_{i}\right)\right\|$ will be very small due to the localization property of Gaussian RBFNN. From the adaptation law (Equation 13) with $W_{i}^{k}\left(0\right)=0$ , it can be observed that these small values of $S_{\bar{\zeta },k,i}\left(Z_{i}\right)$ will only minimally activate the adaptation of the associated neural weights $W_{\bar{\zeta },k,i}$ . As a result, both $W_{\bar{\zeta },k,i}$ and $W_{\bar{\zeta },k,i}^{T}S_{\bar{\zeta },k,i}\left(Z_{i}\right)$ , as well as ${\bar{W}}_{\bar{\zeta },k,i}$ and ${\bar{W}}_{\bar{\zeta },k,i}^{T}S_{\bar{\zeta },k,i}\left(Z_{i}\right)$ , will remain very small for all i∈I[1,N] , k∈I[1,3] along the periodic trajectory $\phi_{\zeta ,i}\left(Z_{i}\left(t\right)\right){|}_{t\geq T_{i}}$ . This indicates that the entire RBFNN $W_{k,i}^{T}S_{k,i}\left(Z_{i}\right)$ and ${\bar{W}}_{k,i}^{T}S_{k,i}\left(Z_{i}\right)$ can be used to accurately approximate the unknown function $f_{k,i}\left(Z_{i}\right)$ locally along the periodic trajectory $\phi_{\zeta ,i}\left(Z_{i}\left(t\right)\right){|}_{t\geq T_{i}}$ , meaning that.

$$f_{k,i}\left(Z_{i}\right)={\hat{W}}_{\zeta ,k,i}^{T}S_{\zeta ,k,i}\left(Z_{i}\right)+\epsilon_{\zeta_{1},k,i}={\hat{W}}_{k,i}^{T}S_{k,i}\left(Z_{i}\right)+\epsilon_{1,k,i}$$

$$={\bar{W}}_{\zeta ,k,i}^{T}S_{\zeta ,k,i}\left(Z_{i}\right)+\epsilon_{\zeta_{2},k,i}={\bar{W}}_{k,i}^{T}S_{k,i}\left(Z_{i}\right)+\epsilon_{2,k,i},$$

with the approximation accuracy level of $\epsilon_{1,k,i}=\epsilon_{\zeta_{1},k,i}-W_{\bar{\zeta },k,i}^{T}S_{\bar{\zeta },k,i}\left(Z_{i}\right)=O\left(\epsilon_{\zeta_{1},k,i}\right)=O\left(\epsilon_{k,i}\right)$ and $\epsilon_{2,k,i}=\epsilon_{\zeta_{2},k,i}-{\bar{W}}_{\bar{\zeta },k,i}^{T}S_{\bar{\zeta },k,i}\left(Z_{i}\right)=O\left(\epsilon_{\zeta_{2},k,i}\right)=O\left(\epsilon_{k,i}\right)$ for all i∈I[1,N] , k∈I[1,3] . This ends the proof.

Remark 7 The key idea in the proof of Theorem 4 is inspired by Wang and Hill (2018). For more detailed analysis on the learning performance, including quantitative analysis on the learning accuracy levels $\epsilon_{1,i,k}$ and $\epsilon_{2,i,k}$ as well as the learning speed, please refer to Yuan and Wang (2011). Furthermore, the AUV nonlinear dynamics (Equation 9) to be identified do not contain any time-varying random disturbances. This is important to ensure accurate identification/learning performance under the deterministic learning framework. To understand the effects of time-varying external disturbances on deterministic learning performance, interested readers are referred to Yuan and Wang (2012) for more details.

Remark 8 Based on Equation 18, to obtain the constant RBFNN weights ${\bar{W}}_{k,i}$ for all i∈I[1,N] , k∈I[1,3] , one needs to implement the formation learning control law Equations 13, 14 first. Then, according to Theorem 4, after a finite-time transient process, the RBFNN weights $W_{k,i}$ will converge to constant steady-state values. Thus, one can select a time segment $\left[t_{a,i},t_{b,i}\right]$ with $t_{b,i}>t_{a,i}>T_{i}$ for all i∈I[1,N] to record and store the RBFNN weights $W_{k,i}\left(t\right)$ for $t\in \left[t_{a,i},t_{b,i}\right]$ . Finally, based on these recorded data, ${\bar{W}}_{k,i}$ can be calculated off-line using Equation 18.

Remark 9 It is shown in Theorem 4 that locally accurate learning of each individual AUV’s nonlinear uncertain dynamics can be achieved using localized RBFNNs along the periodic trajectory $\phi_{\zeta ,i}\left(Z_{i}\left(t\right)\right){|}_{t\geq T_{i}}$ . The learned knowledge can be further represented and stored in a time-invariant fashion using constant RBFNN, i.e., ${\bar{W}}_{k,i}^{T}S_{k,i}\left(Z_{i}\right)$ for all i∈I[1,N] , k∈I[1,3] . In contrast to many existing techniques (e.g., Peng et al., 2017; Peng et al., 2015), this is the first time, to the authors’ best knowledge, that locally accurate identification and knowledge representation using constant RBFNN are accomplished and rigorously analyzed for multi-AUV formation control under complete uncertain dynamics.

## 5 Formation control with pre-learned dynamics

In this section, we will further address objective 2 of Problem 1, which involves achieving formation control without readapting to the AUV’s nonlinear uncertain dynamics. To this end, consider the multiple AUV systems (Equation 1) and the virtual leader dynamics (Equation 2). We employ the estimator observer Equations 3, 4 to cooperatively estimate the leader’s state information. Instead of using the DDL feedback control law (Equation 13), and self-adaptation law (Equation 4), we introduce the following constant RBFNN controller, which does not require online adaptation of the NN weights:

$$\tau_{i}=-J_{i}^{T}\left(\eta_{i}\right)z_{1,i}-K_{2,i}z_{2,i}+{\bar{W}}_{i}^{T}S_{i}^{F}\left(Z_{i}\right),$$ (20)

where ${\bar{W}}_{i}^{T}S_{i}^{F}\left(Z_{i}\right)={\left[{\bar{W}}_{1,i}^{T}S_{1,i}\left(Z_{i}\right),{\bar{W}}_{2,i}^{T}S_{2,i}\left(Z_{i}\right),{\bar{W}}_{3,i}^{T}S_{3,i}\left(Z_{i}\right)\right]}^{T}$ is obtained from Equation 18. The term ${\bar{W}}_{k,i}^{T}S_{k,i}\left(Z_{i}\right)$ represents the locally accurate RBFNN approximation of the nonlinear uncertain function $f_{k,i}\left(Z_{i}\right)$ along the trajectory $\phi_{\zeta ,i}\left(Z_{i}\left(t\right)\right){|}_{t\geq T_{i}}$ , and the associated constant neural weights ${\bar{W}}_{k,i}$ are obtained from the formation learning control process as discussed in Remark 8.

Theorem 5 Consider the multi-AUV system (Equation 1) and the virtual leader dynamics (Equation 3) with the network communication topology G . Under Assumptions 1, 2, the formation control performance (i.e., $\eta_{i}$ converges to $\eta_{0}+d_{i}^{*}$ exponentially with the same $\eta_{0}$ and $d_{i}^{*}$ defined in Theorem 3 for all i∈I[1,N] ) can be achieved by using the DA observer Equations 3, 20 and the constant RBFNN control law (Equation 4) with the constant NN weights obtained from Equation 18.

Proof: The closed-loop system for each local AUV agent can be established by integrating the controller (Equation 20) with the AUV dynamics (Equation 1).

$$\begin{array}{cc} {\dot{z}}_{1,i}= & -K_{1,i}z_{1,i}+J_{i}\left(\eta_{i}\right)z_{2,i},\\ {\dot{z}}_{2,i}= & M_{i}^{-1}\left(-J_{i}^{T}\left(\eta_{i}\right)z_{1,i}-K_{2,i}z_{2,i}+{\bar{W}}_{i}^{T}S_{i}^{F}\left(Z_{i}\right)-F_{i}\left(Z_{i}\right)\right)\\ = & M_{i}^{-1}\left(-J_{i}^{T}\left(\eta_{i}\right)z_{1,i}-K_{2,i}z_{2,i}-\epsilon_{2,i}\right),\;\forall i\in I\left[1,N\right], \end{array}$$

where $\epsilon_{2,i}={\left[\epsilon_{21,i},\epsilon_{22,i},\epsilon_{23,i}\right]}^{T}$ . Consider the Lyapunov function candidate $V_{z,i}=\frac{1}{2}z_{1,i}^{T}z_{1,i}+\frac{1}{2}z_{2,i}^{T}M_{i}z_{2,i}$ , whose derivative along the closed-loop system described is given by:

$$\begin{array}{cc} {\dot{V}}_{z,i}= & z_{1,i}^{T}\left(-K_{1,i}z_{1,i}+J_{i}\left(\eta_{i}\right)z_{2,i}\right)+z_{2,i}^{T}\left(-J_{i}^{T}\left(\eta_{i}\right)z_{1,i}-K_{2,i}z_{2,i}-\epsilon_{2,i}\right)\\ = & -z_{1,i}^{T}K_{1,i}z_{1,i}-z_{2,i}^{T}K_{2,i}z_{2,i}-z_{2,i}^{T}\epsilon_{2,i}. \end{array}$$

Selecting $K_{2,i}=K_{1,i}+K_{22,i}$ where $K_{1,i},K_{22,i}\in S_{3}^{+}$ , we can utilize the method of completing squares to obtain:

$$-z_{2,i}^{T}K_{22,i}z_{2,i}-z_{2,i}^{T}\epsilon_{2,i}\leq \left(\frac{\|\epsilon_{2,i}{\|}^{2}}{4\underline{\lambda }\left(K_{22,i}\right)}\right)\leq \left(\frac{\|\epsilon_{2,i}^{*}{\|}^{2}}{4\underline{\lambda }\left(K_{22,i}\right)}\right),$$

which implies that:

$${\dot{V}}_{z,i}\leq -z_{1,i}^{T}K_{1,i}z_{1,i}-z_{2,i}^{T}K_{1,i}z_{2,i}+\left(\frac{\|\epsilon_{2,i}^{*}{\|}^{2}}{4\underline{\lambda }\left(K_{22,i}\right)}\right)\leq -\rho_{i}V_{z,i}+\delta_{i},\;\forall i\in I\left[1,N\right].$$

where $\rho_{i}=\min\left\{2\underline{\lambda }\left(K_{1,i}\right),\left(2\underline{\lambda }\left(K_{1,i}\right)/\bar{\lambda }\left(M_{i}\right)\right)\right\}$ and $\delta_{i}=\left(\|\epsilon_{2,i}^{*}{\|}^{2}/4\underline{\lambda }\left(K_{22,i}\right)\right)$ . Using similar reasoning to that in the proof of Theorem 2, it is evident from the derived inequality that all signals within the closed-loop system remain bounded. Additionally, $\eta_{i}-\eta_{i}^{d}$ will converge to a small neighborhood around zero within a finite period. The magnitude of this neighborhood can be minimized by appropriately choosing large values for $\underline{\lambda }\left(K_{1,i}\right)>0$ and $\underline{\lambda }\left(K_{2,i}\right)>\bar{\lambda }\left(K_{1,i}\right)$ across all i∈I[1,N] . In line with Theorem 1, under Assumptions 1, 2, the implementation of the DA observer DA observer Equations 3, 4 facilitates the exponential convergence of $\eta_{i}$ towards $\eta_{0}$ . This conjunction of factors assures that $\eta_{i}$ rapidly aligns with $\eta_{d,i}=\eta_{0}+d_{i}^{*}$ , achieving the objectives set out for formation control.

Remark 10 Building on the locally accurate learning outcomes discussed in Section 4, the newly developed distributed control protocol comprising Equations 3, 4, 20 facilitates stable formation control across a repeated formation pattern. Unlike the formation learning control approach outlined in Section 3.2, which involves Equations 3, 4 coupled with Equations 13, 14, the current method eliminates the need for online RBFNN adaptation for all AUV agents. This significantly reduces the computational demands, thereby enhancing the practicality of implementing the proposed distributed RBFNN formation control protocol. This innovation marks a significant advancement over many existing techniques in the field.

## 6 Simulation

We consider a multi-AUV heterogeneous system composed of 5 AUVs for the simulation. The dynamics of these AUVs are described in THE system (Equation 1). The system parameters for each AUV are specified as follows:

$$\begin{array}{cc} M_{i} & =\left[\begin{array}{ccc} m_{11,i} & 0 & 0\\ 0 & m_{22,i} & m_{23,i}\\ 0 & m_{23,i} & m_{33,i} \end{array}\right],\\ C_{i} & =\left[\begin{array}{ccc} 0 & 0 & -m_{22,i}v_{i}-m_{23,i}r_{i}\\ 0 & 0 & -m_{11,i}u_{i}\\ m_{22,i}v_{i}+m_{23,i}r_{i} & -m_{11,i}u_{i} & 0 \end{array}\right],\\ D_{i} & =\left[\begin{array}{ccc} d_{11,i}\left(\nu_{i}\right) & 0 & 0\\ 0 & d_{22,i}\left(\nu_{i}\right) & d_{23,i}\left(\nu_{i}\right)\\ 0 & d_{32,i}\left(\nu_{i}\right) & d_{33,i}\left(\nu_{i}\right) \end{array}\right],\;g_{i}=0,\\ \Delta_{i} & =\left[\begin{array}{c} \Delta_{1,i}\left(\chi_{i}\right)\\ \Delta_{2,i}\left(\chi_{i}\right)\\ \Delta_{3,i}\left(\chi_{i}\right) \end{array}\right],\;\forall i\in I\left[1,5\right], \end{array}$$

where the mass and damping matrix components for each AUV i are defined as:

$$\begin{array}{llll} m_{11,i} & =m_{i}-X_{\dot{u},i}, & m_{22,i} & =m_{i}-Y_{\dot{v},i},\\ m_{23,i} & =m_{i}x_{g,i}-Y_{\dot{r},i}, & m_{33,i} & =I_{z,i}-N_{\dot{r},i},\\ d_{11,i} & =-\left(X_{u,i}+X_{uu,i}\|u_{i}\|\right), & d_{22,i} & =-\left(Y_{v,i}+Y_{vv,i}\|v_{i}\|+Y_{rv,i}\|r_{i}\|\right),\\ d_{23,i} & =-\left(Y_{r,i}+Y_{vr,i}\|v_{i}\|+Y_{rr,i}\|r_{i}\|\right), & d_{32,i} & =-\left(N_{v,i}+N_{vv,i}\|v_{i}\|+N_{rv,i}\|r_{i}\|\right),\\ d_{33,i} & =-\left(N_{r,i}+N_{vr,i}\|v_{i}\|+N_{rr,i}\|r_{i}\|\right). & \end{array}$$

According to the notations in Prestero (2001) and Skjetne et al. (2005) the coefficients {X(⋅),Y(⋅),N(⋅)} are hydrodynamic parameters. For the associated system parameters are borrowed from Skjetne et al. (2005) (with slight modifications for different AUV agents) and simulation purposes and listed in Table 1. For all i∈I[1,5] , we set $x_{g,i}=0.05$ and $Y_{\dot{r},i}=Y_{rv,i}=Y_{vr,i}=Y_{rr,i}=N_{rv,i}=N_{rr,i}=N_{vv,i}=N_{vr,i}=N_{r,i}=0$ . Model uncertainties are given by:

$$\begin{array}{ll} \Delta_{1} & =0,\;\Delta_{2}={\left[\begin{array}{ccc} 0.2u_{2}^{2}+0.3v_{2} & -0.95 & 0.33\|r_{2}\| \end{array}\right]}^{T}\\ \Delta_{3} & ={\left[\begin{array}{ccc} -0.58+\cos\left(v_{3}\right) & 0.23r_{3}^{3} & 0.74u_{3}^{2} \end{array}\right]}^{T}\\ \Delta_{4} & ={\left[\begin{array}{ccc} -0.31 & 0 & 0.38u_{4}^{2}+v_{4}^{3} \end{array}\right]}^{T}\\ \Delta_{5} & ={\left[\begin{array}{ccc} \sin\left(v_{5}\right) & \cos\left(u_{5}+r_{5}\right) & -0.65 \end{array}\right]}^{T}. \end{array}$$

**TABLE 1:** Parameters of AUVs.

Parameter	AUV 1	AUV 2	AUV 3	AUV 4	AUV 5
m (kg)	23	25	20	30	35
$I_{z}$ (kg $\cdot {\text{m}}^{2}$ )	1.8	2.0	1.5	2.2	2.5
$X_{\dot{u}}$ (kg)	−2.0	−2.5	−1.5	−2.5	−3.0
$Y_{\dot{v}}$ (kg)	−10	−10	−10	−15	−15
$N_{\dot{r}}$ (kg $\cdot {\text{m}}^{2}$ )	−1.0	−1.5	−1.0	−2.5	−2.5
$X_{u}$ (kg/s)	−0.8	−1.0	−1.0	−1.5	−2.0
$Y_{v}$ (kg/s)	−0.9	−1.0	−0.8	−1.5	−1.5
$Y_{r}$ (kg/s)	0.1	0.2	0.1	0.2	0.5
$N_{v}$ (kg $\cdot {\text{m}}^{2}$ /s)	0.1	0.1	0.05	0.3	0.35
$X_{uu}$ (kg/m)	−1.3	−1.3	−1.0	−0.85	−1.5
$Y_{vv}$ (kg/m)	−36	−25	−20	−15	−20

**FIGURE 2:**
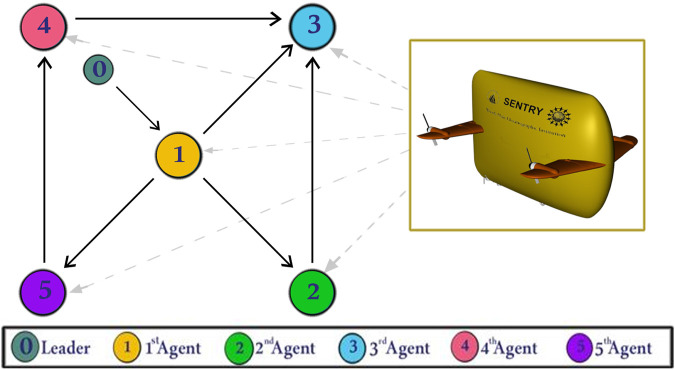
The communication network topology and spanning tree of multi-AUV system of the simulation with 0 as virtual leader.

Figure 2 illustrates the communication topology and the spanning tree where agent 0 is the virtual leader and is considered as the root, in accordance with Assumption 2. The desired formation pattern requires each AUV, $\eta_{i}$ , to track a periodic signal generated by the virtual leader $\eta_{0}$ . The dynamics of the leader are defined as follows:

$$\begin{array}{cc} \left[\begin{array}{c} {\dot{\eta }}_{0}\\ {\dot{\nu }}_{0} \end{array}\right]= & \left[\begin{array}{cc} 0 & \left[\begin{array}{ccc} 1 & 0 & 0\\ 0 & -1 & 0\\ 0 & 0 & 1 \end{array}\right]\\ \left[\begin{array}{ccc} -1 & 0 & 0\\ 0 & 1 & 0\\ 0 & 0 & -1 \end{array}\right] & 0 \end{array}\right]\left[\begin{array}{c} \eta_{0}\\ \nu_{0} \end{array}\right],\\ \left[\begin{array}{c} \eta_{0}\left(0\right)\\ \nu_{0}\left(0\right) \end{array}\right]= & {\left[\begin{array}{cccccc} 0 & 80 & 0 & 80 & 0 & 80 \end{array}\right]}^{T}. \end{array}$$ (21)

The initial conditions and system matrix are structured to ensure all eigenvalues of $A_{0}$ lie on the imaginary axis, thus satisfying Assumption 1. The reference trajectory for $\eta_{0}$ is defined as ${\left[80\sin\left(t\right),80\cos\left(t\right),80\sin\left(t\right)\right]}^{T}$ . The predefined offsets $d_{i}^{*}$ , which determine the relative positions of the AUVs to the leader, are specified as follows:

$$d_{1}^{*}={\left[0,0,0\right]}^{T},d_{4}^{*}={\left[-10,10,0\right]}^{T},d_{2}^{*}={\left[10,-10,0\right]}^{T},d_{5}^{*}={\left[-10,-10,0\right]}^{T},d_{3}^{*}={\left[10,10,0\right]}^{T}.$$

Each AUV tracks its respective position in the formation by adjusting its location to $\eta_{i}=\eta_{0}+d_{i}^{*}$ .

### 6.1 DDL formation learning control simulation

The estimated virtual leader’s state, derived from the cooperative estimator in the first layer (see Equations 3, 4), is utilized to estimate each agent’s complete uncertain dynamics within the DDL controller (second layer) using Equations 13, 14. The uncertain nonlinear functions $F_{i}\left(Z_{i}\right)$ for each agent are approximated using RBFNN, as described in Equation 11. Specifically, for each agent i∈{1,…,5} , the nonlinear uncertain functions $F_{i}\left(Z_{i}\right)$ , dependent on $\nu_{i}$ , are modeled. The input to the NN, $Z_{i}={\left[u_{i},v_{i},r_{i}\right]}^{T}$ , allows the construction of Gaussian RBFNN, represented by $W_{k,i}^{T}S_{k,i}\left(Z_{i}\right)$ , utilizing 4,096 neurons arranged in an 16×16×16 grid. The centers of these neurons are evenly distributed over the state space [−100,100]×[−100,100]×[−100,100] , and each has a width $\gamma_{k,i}=60$ , ensuring bounded and structured parameter optimization for all i∈{1,…,5} and k∈{1,2,3} .

The observer and controller parameters are chosen as $\beta_{1}=\beta_{2}=5$ , and the diagonal matrices $K_{1,i}=800*\text{diag}\left\{1.2,1,1\right\}$ and $K_{2,i}=1200*\text{diag}\left\{1.2,1,1\right\}$ , with $\Gamma_{k,i}=10$ and $\sigma_{k,i}=0.0001$ for all i∈{1,…,5} and k∈{1,2,3} . The initial conditions for the agents are set as $\eta_{1}\left(0\right)={\left[30,60,0\right]}^{T}$ , $\eta_{2}\left(0\right)={\left[40,70,0\right]}^{T}$ , $\eta_{3}\left(0\right)={\left[50,80,0\right]}^{T}$ , $\eta_{4}\left(0\right)={\left[10,70,0\right]}^{T}$ , and $\eta_{5}\left(0\right)={\left[10,50,0\right]}^{T}$ . Zero initial conditions are assumed for all the distributed observer states $\left(\chi_{i,0},A_{i,0}\right)$ and the DDL controller states $W_{k,i}$ for all i∈{1,…,5} and k∈{1,2,3} . Time-domain simulation is carried out using the DDL formation learning control laws as specified in Equations 13, 14, along with Equations 3, 4.

**FIGURE 3:**
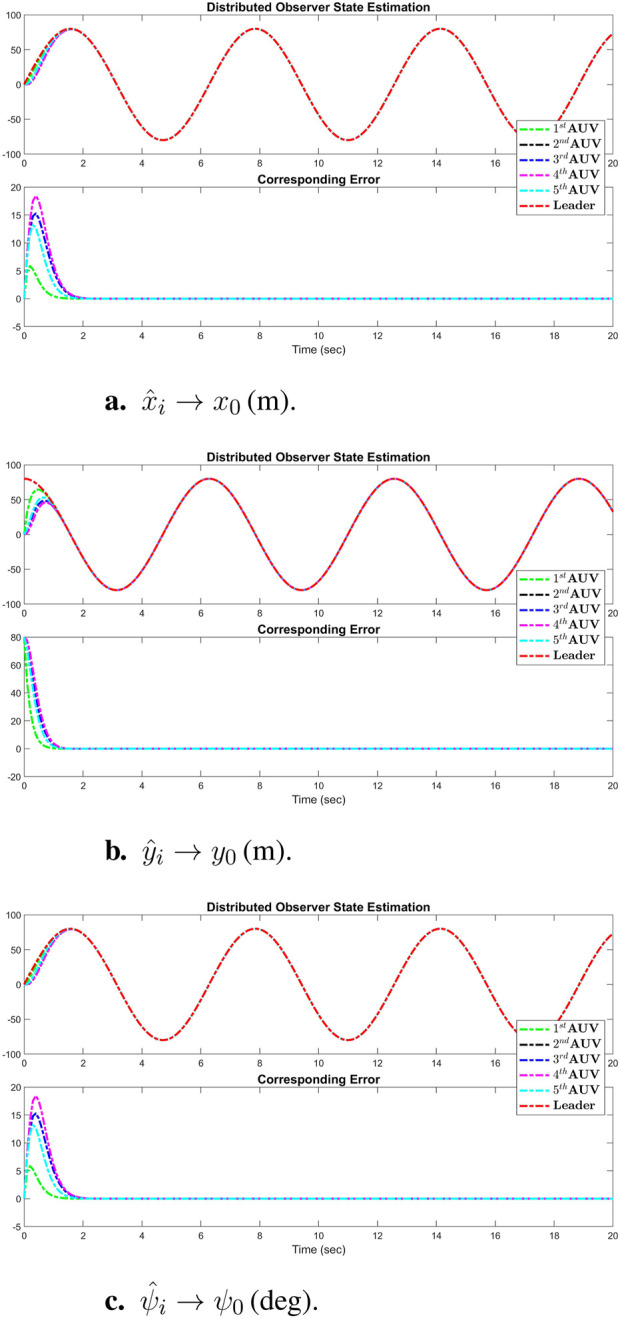
Simulation results of the cooperative observer (first layer) for all three states (x-axis, y-axis, and vehicle heading) of each AUV: **(A)** ${\hat{x}}_{i}\to x_{0}\;\left(\text{m}\right)$ , **(B)** ${\hat{y}}_{i}\to y_{0}\;\left(\text{m}\right)$ , **(C)** ${\hat{\psi }}_{i}\to \psi_{0}\;\left(\text{deg}\right)$ .

**FIGURE 4:**
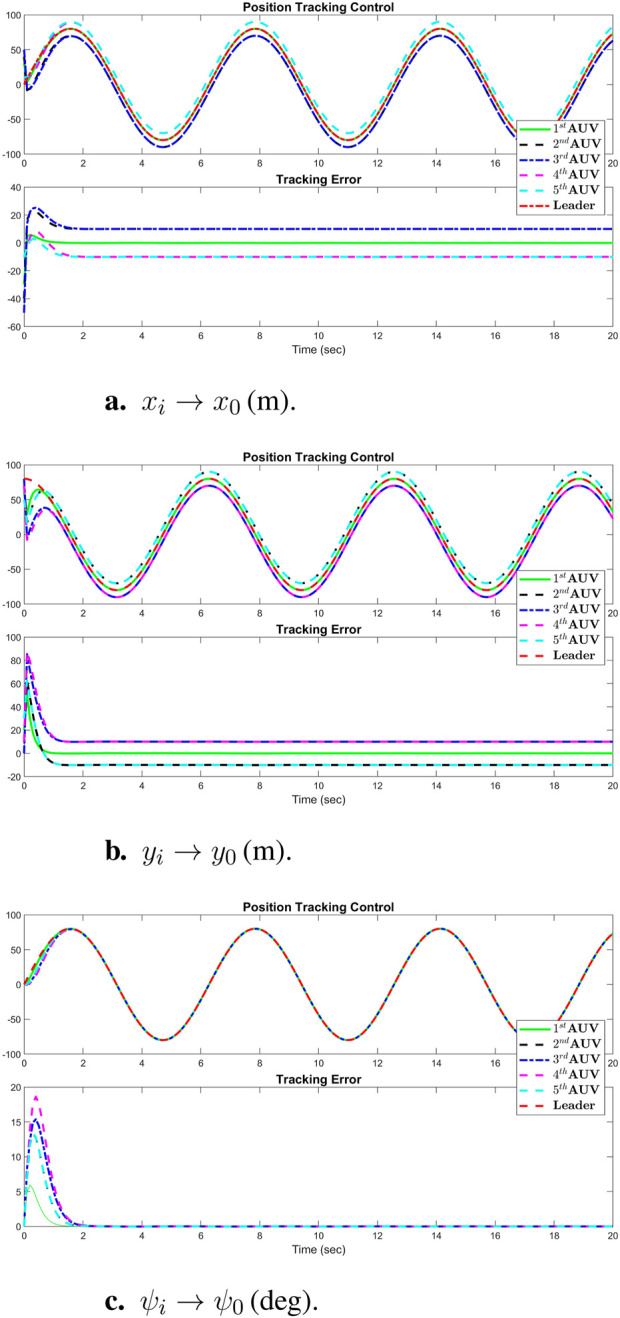
Simulation results of position tracking control performance of all agents: **(A)** $x_{i}\to x_{0}\;\left(\text{m}\right)$ , **(B)** $y_{i}\to y_{0}\;\left(\text{m}\right)$ , **(C)** $\psi_{i}\to \psi_{0}\;\left(\text{deg}\right)$ .

**FIGURE 5:**
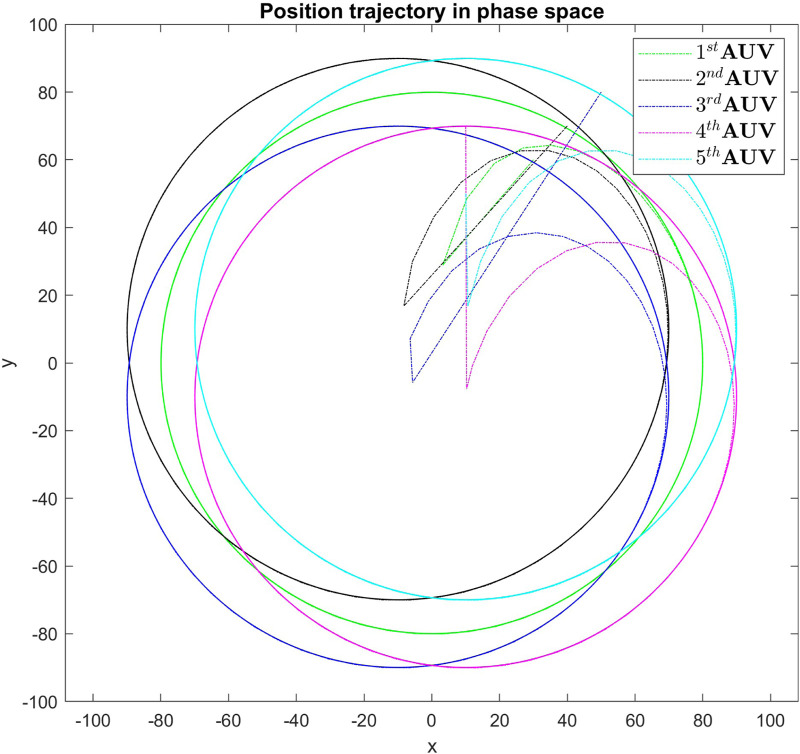
Real-time control performance in simulation for all agents, demonstrating the tracking strategy’s effectiveness in maintaining the formation pattern.

Figure 3 displays the simulation results of the cooperative estimator (first layer) for all five agents. It illustrates how each agent’s estimated states, ${\hat{\eta }}_{i}$ , converge perfectly to the leader’s states ${\hat{\eta }}_{0}$ thorough Equations 3, 4. Figure 4 presents the position tracking control responses of all agents. Figures 4A–C illustrate the tracking performance of AUVs along the *x*-axis, *y*-axis, and vehicle heading, respectively, demonstrating effective tracking of the leader’s position signal. While the first AUV exactly tracks the leader’s states, agents 2 through 5 are shown to successfully follow agent 1, maintaining prescribed distances and alignment along the *x* and *y* axes, and matching the same heading angle. These results underscore the robustness of the real-time tracking control system, which enforces a predefined formation pattern, initially depicted in Figure 2. Additionally, Figure 5 highlights the real-time control performance for all agents, showcasing the effectiveness of the tracking strategy in maintaining the formation pattern.

**FIGURE 6:**
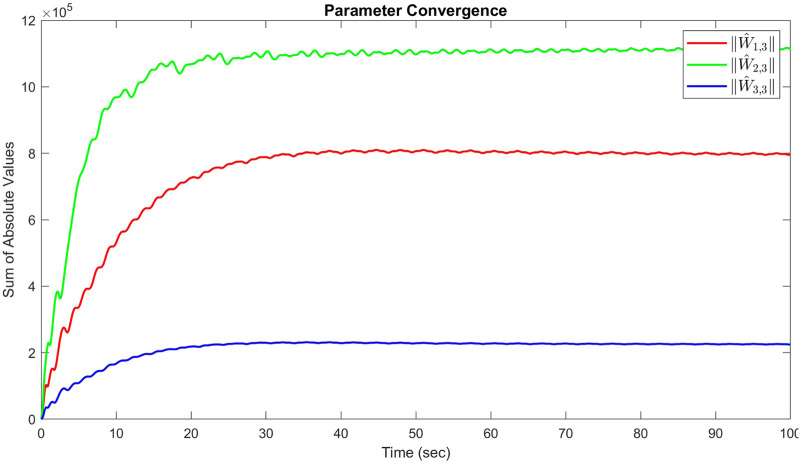
Sum of the absolute values of neural network weights in simulation for the third agent, showing the network stabilized and learns a consistent tracking pattern.

**FIGURE 7:**
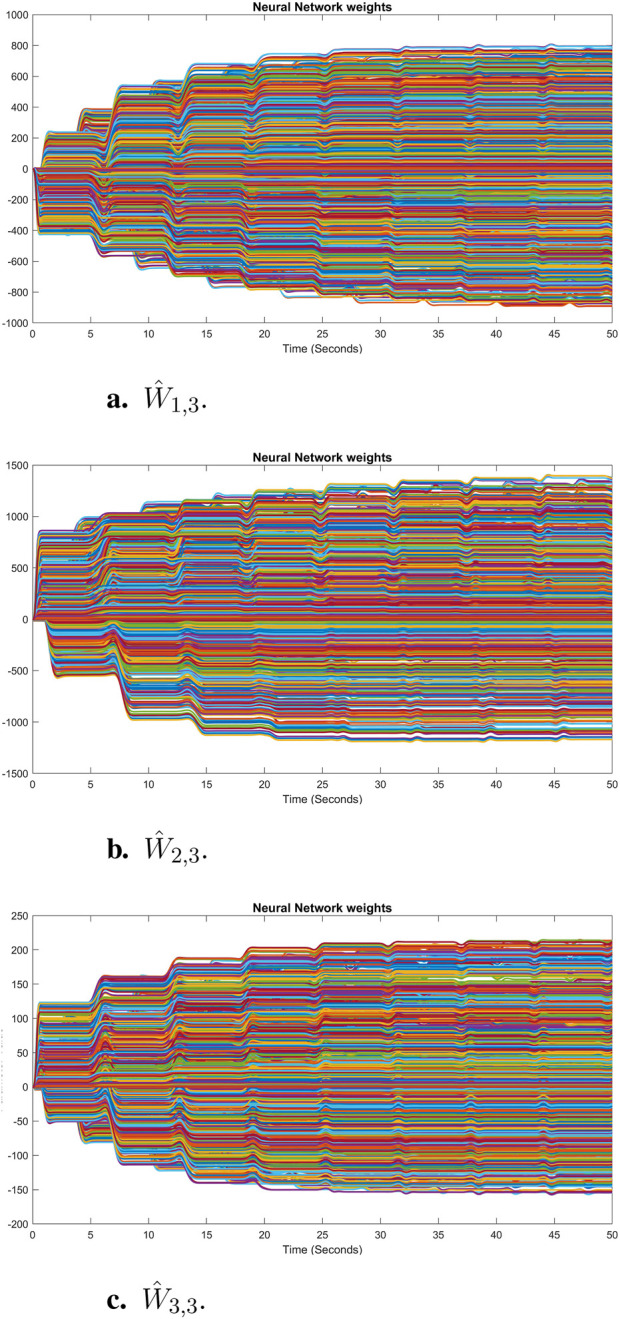
Convergence of neural network weights of each state to their optimal values in simulation for the third agent: **(A)** ${\hat{W}}_{1,3}$ , **(B)** ${\hat{W}}_{2,3}$ , **(C)** ${\hat{W}}_{3,3}$ . The stabilized weights demonstrate accurate learning in the second layer throughout the training process.

**FIGURE 8:**
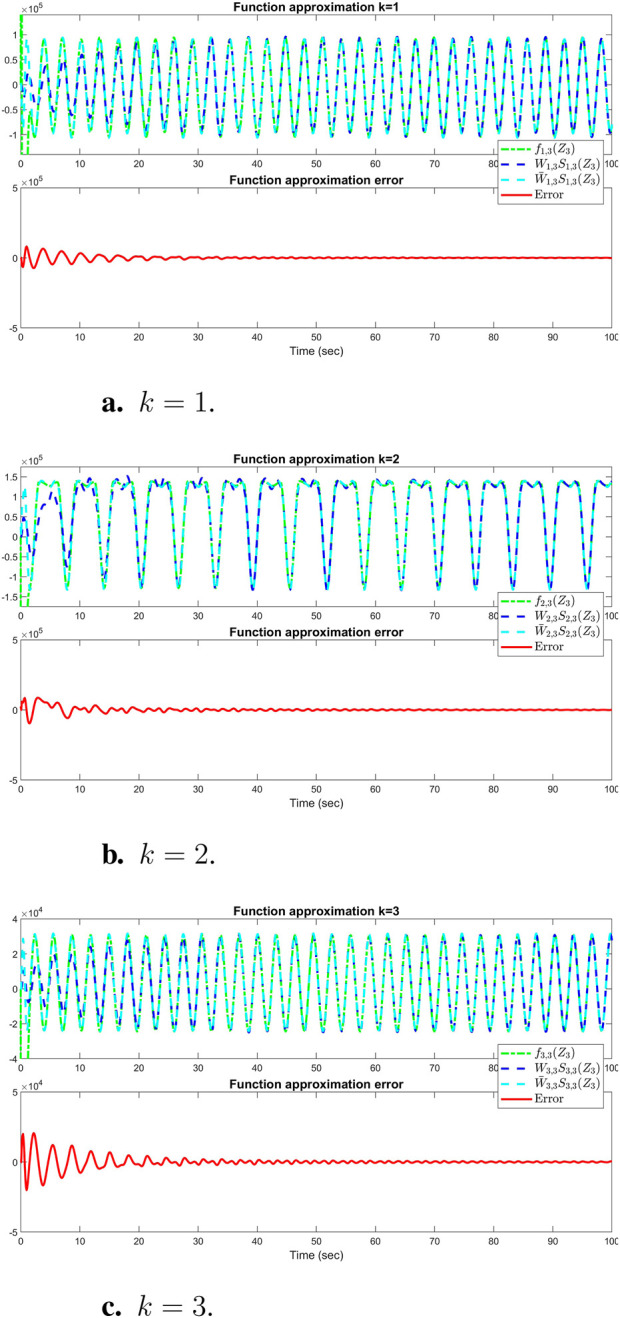
Simulation results of successful function approximation for all three states (k = 1, 2, 3) of the $3^{\mathrm{rd}}$ AUV: **(A)** k=1 , **(B)** k=2 , **(C)** k=3 . Comparison of $f_{k,3}\left(Z_{3}\right)$ , $W_{k,3}^{T}S_{k,3}\left(Z_{3}\right)$ , and ${\bar{W}}_{k,3}^{T}S_{k,3}\left(Z_{3}\right)$ using stored constant NN weights $\left({\bar{W}}_{k,3}\right)$ .

The sum of the absolute values of the neural network weights in Figure 6. This convergence reflects the network’s ability to maintain consistent performance, as further adjustments to the weights become minimal. Also, updating neural network weights and their convergence throughout the learning process into their optimal valies depicted in Figure 7. This convergence of all neural network weights to their optimal values during the training process, aligns with Theorem 4 as well. This leads to achieving accurate function approximation in the second layer. Figure 8 represents the successful function approximation results for the unknown system dynamics $F_{3}\left(Z_{3}\right)$ as defined in Equation 11 for the third AUV, using RBFNN. The approximations are plotted for both $W_{k,3}^{T}S_{k,3}\left(Z_{3}\right)$ and ${\bar{W}}_{k,3}^{T}S_{k,3}\left(Z_{3}\right)$ for all k∈I[1,3] which defined in Theorem 4. The results confirm that locally accurate approximations of the AUV’s nonlinear dynamics were achieved. Moreover, this learned knowledge about the dynamics is effectively stored and represented using localized constant RBFNN.

### 6.2 Simulation for formation control with pre-learned dynamics

**FIGURE 9:**
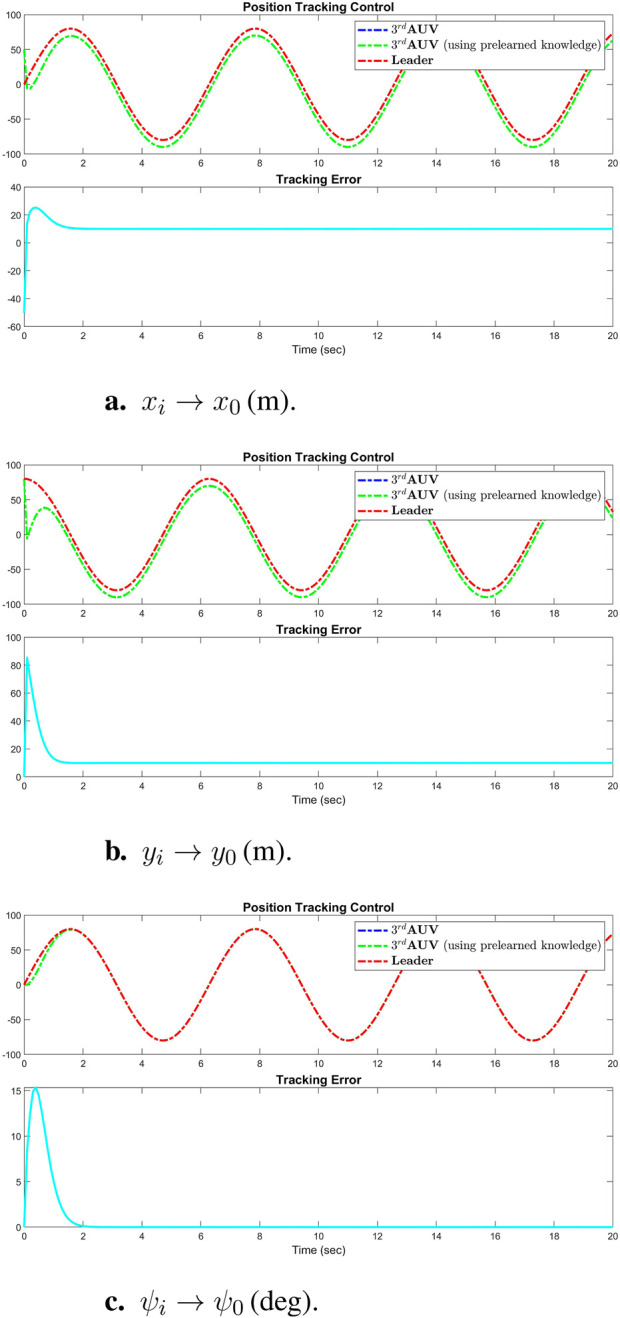
Simulation results of successful performance of position tracking control using pretrained weights $\left(\bar{W}\right)$ : **(A)** $x_{i}\to x_{0}\;\left(\text{m}\right)$ , **(B)** $y_{i}\to y_{0}\;\left(\text{m}\right)$ , **(C)** $\psi_{i}\to \psi_{0}\;\left(\text{deg}\right)$ .

To evaluate the distributed control performance of the multi-AUV system, we implemented the pre-learned distributed formation control law. This strategy integrates the estimator observer Equations 3, 4, this time coupled with the constant RBFNN controller (Equation 21). We employed the virtual leader dynamics described in Equation 21 to generate consistent position tracking reference signals, as previously discussed in Section 6.1. To ensure a fair comparison, identical initial conditions and control gains and inputs were used across all simulations. Figure 9 illustrates the comparison of the tracking control results from Equations 13, 14 with the results using pre-trained weights $\bar{W}$ in Equation 20.

The control experiments and simulation results presented demonstrate that the constant RBFNN control law (Equation 4) can achieve satisfactory tracking control performance comparable to that of the adaptive control laws (Equations 13, 14), but with no computational demand. The elimination of online recalculations or readaptations of the NN weights under this control strategy significantly reduces the computational load whenever system restarts without needing to retrain again. This reduction is particularly advantageous in scenarios involving extensive neural networks with a large number of neurons, thereby conserving system energy and enhancing operational efficiency in real-time applications.

Before concluding the paper, a brief contribution of the paper is provided:

• Distributed Observer Results: Simulations showed that the distributed observer effectively estimated the leader’s state, allowing for accurate formation control without needing global information.
• Tracking Control Results: The controller demonstrated reliable tracking of reference signals, maintaining performance even under varying conditions and unknown system dynamics.
• Formation Control: The proposed controller maintained accurate formation control relative to a virtual leader in simulations, even when the system dynamics were unknown with different AUVs.
• Neural Network Weight Convergence: The simulation results demonstrated that the neural network weights converged effectively, ensuring accurate function approximation and reliable performance in controlling AUVs under uncertainties.
• Adaptability and Stability: The framework ensured stable tracking performance across various environmental conditions by relying on the RBFNN’s learning capabilities, allowing the AUVs to use prelearned information and maintain formation control without needing to relearn dynamics whenever system restarts.
• Reduction in Computational Load: The use of pre-trained neural network weights significantly reduced the computational burden during real-time operation, particularly when large neural networks were employed.

## 7 Conclusion

In conclusion, this paper has introduced a novel two-layer control framework designed for Autonomous Underwater Vehicles (AUVs), aimed at universal applicability across various AUV configurations and environmental conditions. This framework assumes all system dynamics to be unknown, thereby enabling the controller to operate independently of specific dynamic parameters and effectively handle any environmental challenges, including hydrodynamic forces and torques. The framework consists of a first-layer distributed observer estimator that captures the leader’s dynamics using information from adjacent agents, and a second-layer decentralized deterministic learning controller. Each AUV utilizes the estimated signals from the first layer to determine the desired trajectory, simultaneously training its own dynamics using Radial Basis Function Neural Networks (RBFNN). This innovative approach not only sustains stability and performance in dynamic and unpredictable environments but also allows AUVs to efficiently utilize previously learned dynamics after system restarts, facilitating rapid resumption of optimal operations. The robustness and versatility of this framework have been rigorously confirmed through comprehensive simulations, demonstrating its potential to significantly enhance the adaptability and resilience of AUV systems. By embracing total uncertainty in system dynamics, this framework establishes a new benchmark in autonomous underwater vehicle control and lays a solid groundwork for future developments aimed at minimizing energy use and maximizing system flexibility. We plan to expand this framework by accommodating more general leader dynamics and conducting experimental applications to validate its performance in real-world settings. Moreover, a more accurate model of some source of uncertainty could improve performance which we will address in our future research. The authors declare that the research was conducted in the absence of any commercial or financial relationships that could be construed as a potential conflict of interest.

## Data Availability

The original contributions presented in the study are included in the article/supplementary material, further inquiries can be directed to the corresponding author.

## Footnotes

1 A recurrent trajectory represents a large set of periodic and periodic-like trajectories generated from linear/nonlinear dynamical systems. A detailed characterization of recurrent trajectories can be found in Wang and Hill (2018).
